# Ni/Co-Catalyzed Homo-Coupling of Alkyl Tosylates

**DOI:** 10.3390/molecules24081458

**Published:** 2019-04-12

**Authors:** Kimihiro Komeyama, Ryusuke Tsunemitsu, Takuya Michiyuki, Hiroto Yoshida, Itaru Osaka

**Affiliations:** Department of Applied Chemistry, Graduate School of Engineering, Hiroshima University, 1-4-1 Kagamiyama, Higashi-Hiroshima City, Hiroshima 739-8527, Japan; m192949@hiroshima-u.ac.jp (R.T.); m186210@hiroshima-u.ac.jp (T.M.); yhiroto@hiroshima-u.ac.jp (H.Y.); iosaka@hiroshima-u.ac.jp (I.O.)

**Keywords:** homo-coupling, S_N_2-type oxidative addition, transalkylation, alkyl tosylates, cobalt catalyst, nickel catalyst

## Abstract

A direct reductive homo-coupling of alkyl tosylates has been developed by employing a combination of nickel and nucleophilic cobalt catalysts. A single-electron-transfer-type oxidative addition is a pivotal process in the well-established nickel-catalyzed coupling of alkyl halides. However, the method cannot be applied to the homo-coupling of ubiquitous alkyl tosylates due to the high-lying σ*(C–O) orbital of the tosylates. This paper describes a Ni/Co-catalyzed protocol for the activation of alkyl tosylates on the construction of alkyl dimers under mild conditions.

## 1. Introduction

**Scheme 1:**
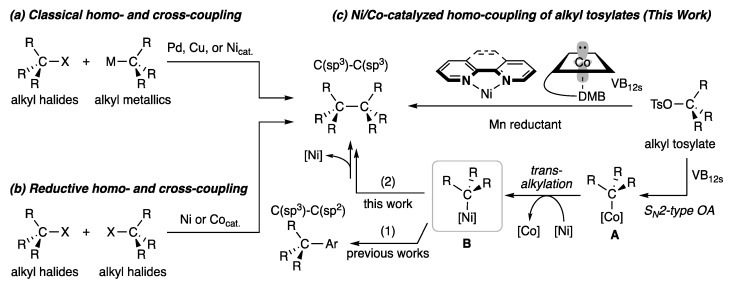
The transition metal-catalyzed C(sp^3^)–C(sp^3^) bond construction.

The development of synthetic methods for carbon–carbon bonds is one of the central challenges in organic synthesis. Particularly, C(sp^3^)–C(sp^3^) linkages are the most abundant carbon skeleton rather than C(sp^3^)–C(sp^2^) and C(sp^2^)–C(sp^2^) linkages in naturally occurring products and pharmaceuticals [1]. In the past few decades, a great advance has been made in the transition-metal-catalyzed C(sp^3^)–C(sp^3^) coupling between alkyl halides and alkyl metallic reagents (alkyl-MgX, -ZnX, and -BR_2_) by Pd [2,3,4], Cu [5,6], and Ni [7,8,9,10,11,12,13] catalysts (Scheme 1a). However, alkyl metallic reagents are generally prepared from the corresponding alkyl halides and are sensitive to polar functional groups. Although alkyl-BR_2_ is stable and available in numerous cross-couplings, they require basic additives to activate for the transmetalation of Alkyl-BR_2_. These inherent reactivities place limitations on synthesizable C(sp^3^)–C(sp^3^) linkages. Therefore, the development of more tractable and practical protocols for the formation of C(sp^3^)–C(sp^3^) linkages without alkyl metallic reagents is still in high demand.

In contrast to the above traditional approaches for the C(sp^3^)–C(sp^3^) linkages, the nickel or cobalt-catalyzed reductive cross- [14,15,16,17,18,19] and homo-coupling [20,21,22,23] between two alkyl halides have been intensively studied over the past decade (Scheme 1b). In these transformations, a single-electron-transfer (SET) process has been adopted for the initial activation step of alkyl halides, enabling a generation of high-valent dialkyl transition-metal intermediates to lead to C(sp^3^)–C(sp^3^) linkages via a rapid reductive elimination without a competitive β-H elimination. Despite recent significant progress on such reductive couplings, their alkyl sources have been limited to alkyl halides. Thereby, accessible C(sp^3^)–C(sp^3^) linkages utilizing the reductive coupling intrinsically depend on the availability of alkyl halides. Compared with alkyl halides, alkyl alcohols are present in a diverse set of natural products and medicines and are upstream raw materials for many alkyl halides. Although the transformation of alcohols via a direct cleavage of the robust C(sp^3^)–O bonds is quite tricky due to their high bond dissociation energy, alcohols can be easily converted into stable but highly electrophilic alkyl tosylates, which work as competent carbon-electrophiles in the copper or nickel-catalyzed couplings with Grignard reagents [24]. However, the C(sp^3^)–C(sp^3^) reductive coupling directly utilizing alkyl tosylates is still challenging [17,21] because alkyl tosylates are inert for the SET process due to the high-lying σ*(C–O) orbital of the tosylates, as demonstrated in numerous Ni-catalyzed couplings [25,26,27,28,29,30,31].

**Scheme 2:**
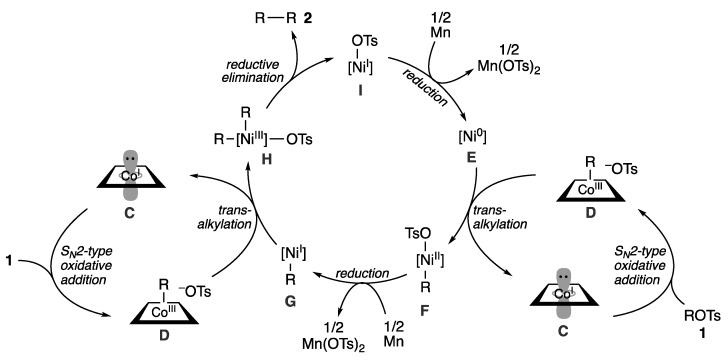
A plausible reaction mechanism of the Ni/Co-catalyzed C(sp^3^)–C(sp^3^) homo-coupling.

Recently, we developed a C(sp^2^)–C(sp^3^) reductive cross-coupling between aryl halides and alkyl tosylates using a combination of nickel and nucleophilic vitamin B_12s_, VB_12s_ (Scheme 1c-1) [32,33]. In the cross-coupling, the cobalt played a crucial role in the activation of alkyl tosylates. Thus, an S_N_2-type oxidative addition of the tosylate to VB_12s_ affords alkyl-cobalt **A**, which could perform a transalkylation with nickel to give an alkyl-nickel **B**, leading to C(sp^3^)–C(sp^2^) linkages in our previous works. It is noteworthy that the alkyl-nickel **B** would also be an intermediate in the homo-coupling. Based on the unique performance of the Ni/Co-hybrid catalyst system, we assumed that the catalyst system might enable a direct homo-coupling of alkyl tosylates to form C(sp^3^)–C(sp^3^) linkages (Scheme 1c-2) without the in situ halogen-OTs exchange [21,34].

## 2. Results and Discussion

### 2.1. Screening of Reaction Conditions

**Figure 1:**
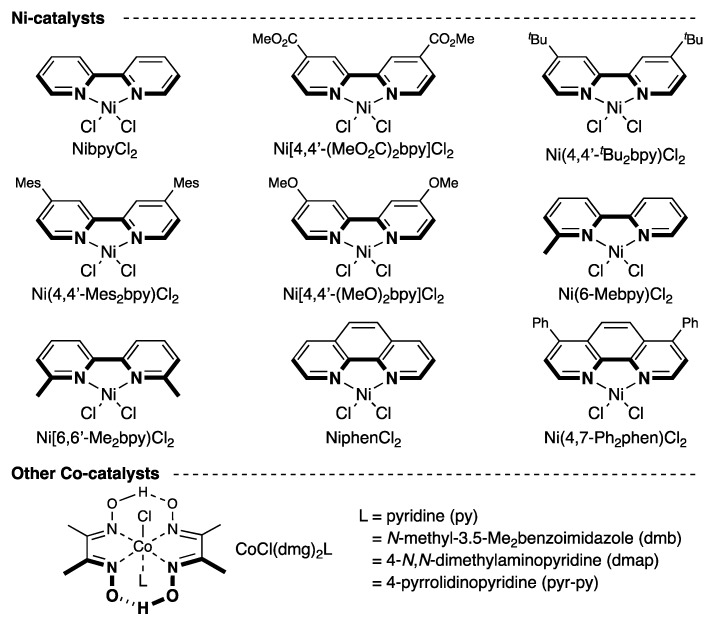
The list of nickel and cobalt catalysts.

To test our hypothesis, we carried out the reaction of 3-(4-anisyl)propyl tosylate (**1a**) as a model substrate using the combination of nickel and cyanocobalamine, VB_12_, catalysts (Table 1). When **1a** was treated with NibpyCl_2_ (10 mol%, bpy = 2,2’-bipyridine, Figure 1) and VB_12_ (10 mol%) in the presence of Mn powder (2.0 equiv.) and DMF (*N*,*N*-dimethylformamide) at 30 °C for 24 h, the alkyl dimer **2a** was obtained in a 72% yield with a complete consumption of the tosylate **1a** (entry 1, Table 1). In the transformation, no detectable amount of β-hydride eliminated and proto-detosylated products of **1a** were obtained (see Supplementary Materials). We carefully confirmed that the lack of nickel and cobalt catalysts did not lead to the dimer **2a** at all (entries 2 and 3), in which most of **1a** remained unchanged after the reaction. These results indicated that alkyl tosylates are not reduced with Mn in the presence or absence of Ni and Co catalysts [35,36,37]. Mn powder is also crucial for efficient homo-coupling; that is, the absence of Mn (entry 4) or the use of Zn instead of Mn (entry 5) caused no reaction or a diminished yield of **2a**, respectively. Additionally, the coupling highly depended on the nickel-ligand. 4,4’-(MeO_2_C)bpy, 4,4’-*^t^*Bu_2_bpy, 4,4’-Mes_2_bpy, 4,4’-(MeO)_2_bpy, 6-Mebpy, and 6,6’-Me_2_bpy (Figure 1) provided **2a** in low yields (entries 6–11). In contrast, phenanthroline-type ligands were effective (entries 12 and 13), particularly 1,10-phen afforded **2a** in a 75% yield (entry 12). Furthermore, we found that the present reaction was sensitive to the solvent; thus DMF (entry 12) and DMSO (dimethyl sulfoxide, entry 14) were superior to THF (tetrahydrofuran), 1,4-dioxane, and acetonitrile (entry 15). The best result (93% yield of **2a**) was accomplished using a NiphenBr_2_ catalyst in DMSO (entry 16). Furthermore, other cobalt complexes like CoCl(dmg)_2_L could also be utilized in the homo-coupling, but these reactions were slow (entry 17).

### 2.2. Substrate Scope

With optimized conditions in hand (entry 16 in Table 1), we next explored the substrate scope in the Ni/Co-catalyzed homo-coupling of alkyl tosylates (Table 2). The homo-coupling tolerated well not only simple alkyl groups (**1b** and **1c**, entries 1 and 2) but also alkenyl and alkynyl substituents (**1d** and **1e**, entries 3 and 4); the corresponding homodimers **2b**–**2e** were provided in good yields. The chloro and pinacolboryl groups on the aryl ring (**1f** and **1g**, entries 5 and 6) did not interfere with the transformation, highlighting the potential of the present coupling in combination with further conventional cross-coupling sequences. Additionally, useful functional groups such as ester (**1h**, entry 7), phthalimide (**1i**, entry 8), and silyl ether (**1j** and **1k**, entries 9 and 10) were compatible in the transformation, giving rise to the corresponding C(sp^3^)–C(sp^3^) linkages in 60–90% yields. Especially, **1k** can be easily synthesized through a regioselective mono-tosylation of the corresponding diol [38,39,40]. Therefore, the homo-coupling **1k** is thought to be of assistance in constructing complex alkyl dimers from polyols. Incidentally, a key step in the homo-coupling would be considered the S_N_2-type oxidative addition of alkyl tosylates to nucleophilic Co(I) to generate alkyl-Co(III) species as shown in Scheme 1 (the formation of the alkyl-cobalt intermediate **A**). Indeed, neighboring substituents at the 2-position of primary alkyl tosylate **1l** and **1m** inhibited the homo-coupling due to the steric repulsion between the substituent and the cobalt center in the transition state in the S_N_2 reaction (entries 11 and 12). Although these couplings required longer reaction time (48–74 h) in a DMF solvent, the corresponding alkyl dimers **2l** and **2m** were obtained in 80% and 65% yield, respectively.

### 2.3. Plausible Reaction Mechanism

**Scheme 3:**
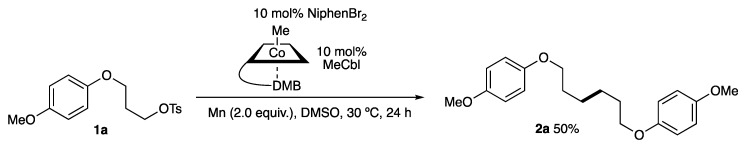
The homo-coupling of **1a** using a combination of NiphenBr_2_ and MeCbl catalysts.

**Scheme 4:**
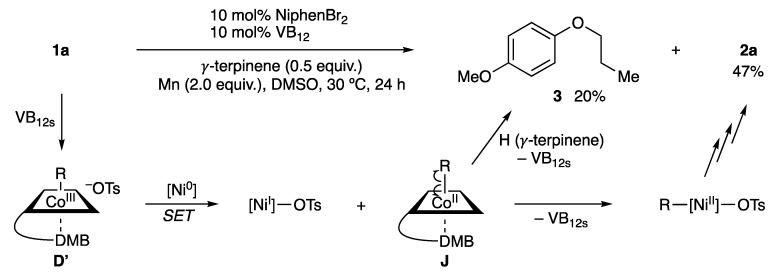
The Ni/Co-catalyzed homo-coupling of **1a** in the presence of γ-terpinene.

Although further mechanistic studies would be needed to understand the present homo-coupling in detail, we propose a plausible reaction mechanism as depicted in Scheme 2. Initially, an S_N_2-type oxidative addition of the alkyl tosylate **1** to the in situ-generated nucleophilic Co(I) species **C** could provide the alkyl-cobalt(III) **D**, followed by transalkylation with the zerovalent nickel **E** to afford alkyl-nickel intermediate **F** [41,42]. The reduction of **F** with Mn gives the monovalent alkyl-nickel intermediate **G**. A second transalkylation between **G** and the alkyl-cobalt(III) **D** provides dialkyl-nickel(III) **H**, which undergoes a rapid reductive elimination to produce the alkyl dimer **2** and the monovalent nickel species **I**. Finally, the catalytic cycle would be closed by a reduction of **I** with Mn to regenerate **E**. As a corroboration of the expected mechanism, the methylcobalamin (MeCbl) catalyst participated in the homo-coupling, leading to the alkyl dimer **2a** in a 50% yield (Scheme 3). The result might imply the formation of the alkyl-cobalt(III) during the reaction. Additionally, the homo-coupling in the presence of hydrogen-atom donor, γ-terpinene (0.5 equiv.), [43] provided the detosyloxylated product **3** in a 20% yield along with the alkyl dimer **2a** in a 47% yield (Scheme 4), indicating the formation of the alkyl radical during the reaction. Thus, a cleavage of the generated alkyl-cobalt(III) **D’** could be induced by an electron transfer from nickel to give the alkyl-cobalt(II) intermediate **J** [41]. Thermodynamically unstable **J** was rapidly converted into alkyl radical and VB_12s_ [44,45]. Most of the radicals were captured by Ni(I)–OTs to produce alkyl dimer **2a**; a part of the alkyl radical could react with γ-terpinene to form the reduction product **3**.

## 3. Materials and Methods

### 3.1. General Information

All reactions were performed on oven- and flame-dried glassware under argon using standard Schlenk techniques. Flash column chromatography was performed with 40–80 nm silica gel 60 (KANTO Chemical Co. Inc., Tokyo, Japan). Analytical thin layer chromatography (TLC) monitoring was carried out with type 60 F_254_ silica gel aluminum sheets (Merck KGaA, Darmstadt, Germany). Gas chromatography (GC) monitoring was carried out on GC-2014 (Shimadzu, Kyoto, Japan) with a 0.25 mm × 60 m TC-1 capillary column (GL Science Co., Torrance, CA, USA). The nuclear magnetic resonance (NMR) spectra were recorded with a Varian-400 (^1^H NMR: 400 MHz; ^13^C NMR: 101 MHz) spectrometer or Varian-500 (^1^H NMR: 500 MHz; ^13^C NMR: 126 MHz) spectrometers (Agilent, Santa Clara, CA, USA), calibrated from residual chloroform and deuterated chloroform as internal standards at 7.26 ppm for ^1^H NMR spectra and at 77.0 ppm for ^13^C NMR spectra, respectively. The high-resolution mass spectrum (HRMS) was performed by the Natural Science Center for Basic Research and Development (N-BARD) of Hiroshima University (Higashi-Hiroshima, Japan) using LTQ Orbitrap XL from (Thermo Fisher Scientific, Waltham, MA, USA). All nickel catalysts were synthesized based on the literature [46]. CoCl(dmgH)_2_L were prepared according to the literature [47]. All solvents and TMSCl were dried over activated Molecular Sieves (MS) 4Å and distilled and stored with activated MS 4Å under argon. All alkyl tosylates were prepared from the corresponding alcohols by the reported methods [48]. Unless otherwise noted, commercially available reagents were used as received without further purification.

### 3.2. General Procedure of the NiBr_2_phen/VB_12_-Catalyzed Homo-Coupling of Alkyl Tosylates

In an oven-dried Pyrex-Schlenk tube, Mn powder (27.5 mg, 0.5 mmol) was added and heated at 400 °C for 5 min under a vacuum to activate the manganese. After cooling, the Schlenk tube was filled with argon. NiphenBr_2_ (10.0 mg, 0.025 mmol). Then, VB_12_ (33.9 mg, 0.025 mmol), DMSO (1.0 mL) and TMSCl (6.4 μL) were added into the tube. After stirring for 10 min at room temperature, the color of the reaction mixture changed from red to black. Alkyl tosylate (0.25 mmol) was added to the reaction mixture and stirred at 30 °C for an appropriate time. The obtained mixture was diluted with ethyl acetate and quenched with saturated aqueous ammonium chloride. At this time, the GC yield was measured using dodecane as an internal standard. The aqueous phase was extracted with ethyl acetate. The combined organic phase was dried over MgSO_4_. After filtration and the removal of the solvent, the residue was purified by a silica-gel column chromatography to get the corresponding alkyl dimer.

### 3.3. Product Characterization

1,6-Di(4-anisyloxy)hexane (**2a**) was isolated as a white solid (Mp.: 78–79 °C) by a silica-gel column chromatography using chloroform as an eluent; ^1^H NMR (400 MHz, CDCl_3_) δ 6.83 (s, 8H), 3.92 (t, *J* = 6.5 Hz, 4H), 3.77 (s, 6H), 1.85–1.74 (m, 4H), 1.59–1.46 (m, 4H); ^13^C NMR (126 MHz, CDCl_3_) δ 153.68, 153.24, 115.42, 114.61, 68.48, 55.72, 29.32, 25.87; HRMS (ESI) calcd for C_20_H_27_O_4_ [M+H]^+^: 331.1909, found: 331.1907.

Tetracosane (**2b**) was isolated as a white solid (Mp.: 45–46 °C) by silica-gel column chromatography using hexane as an eluent; ^1^H NMR (400 MHz, CDCl_3_) δ 1.32–1.23 (m, 44H), 0.88 (t, *J* = 6.7 Hz, 6H); ^13^C NMR (500 MHz, CDCl_3_) δ 31.92, 29.70, 29.36, 22.69, 14.14, 14.10; all peaks were broad or multiplet; HRMS (ESI) calcd for C_24_H_50_: 338.3913, found: 338.3920.

1,8-Diphenyloctane (**2c**) was isolated as a colorless oil by silica-gel column chromatography using a mixture of Hexane and EtOAc (5:1) as an eluent; ^1^H NMR (400 MHz, CDCl_3_) δ 7.32–7.22 (m, 6H), 7.21–7.12 (m, 4H), 2.63–2.55 (m, 4H), 1.60 (dt, *J* = 15.1, 7.4 Hz, 4H), 1.37–1.27 (m, 8H); ^13^C NMR (500 MHz, CDCl_3_) δ 142.90, 128.38, 128.20, 125.53, 35.96, 31.49, 29.41, 29.29; HRMS (ESI) calcd for C_24_H_50_: 266.2035, found: 266.2035.

2,6,11,15-Tetramethylhexadeca-2,14-diene (**2d**) was isolated as a colorless oil by silica-gel column chromatography using hexane as an eluent; ^1^H NMR (500 MHz, CDCl_3_) δ 5.10 (t, *J* = 7.2 Hz, 2H), 2.04–1.87 (m, 4H), 1.68 (s, 6H), 1.60 (s, 6H), 1.44–1.18 (m, 10H), 1.17–1.03 (m, 4H), 0.85 (d, *J* = 6.6 Hz, 6H); ^13^C NMR (500 MHz, CDCl_3_) δ 130.94, 125.10, 37.15, 36.99, 32.39, 27.34, 25.72, 25.57, 19.60, 17.62; HRMS (ESI) calcd for C_20_H_38_: 278.2974, found: 278.2973.

5,11-Hexadecadiyne (**2e**) was isolated as a colorless oil by silica-gel column chromatography using hexane as an eluent; ^1^H NMR (500 MHz, CDCl_3_) δ 2.20–2.10 (m, 8H), 1.62–1.34 (m, 12H), 0.90 (t, *J* = 7.2 Hz, 6H); ^13^C NMR (500 MHz, CDCl_3_) δ 80.44, 79.78, 31.23, 28.24, 21.93, 18.42, 18.32, 13.63; HRMS (ESI) calcd for C_16_H_26_: 218.2035, found: 218.2025.

1,4-Di(4-chlorophenyloxy)hexane (**2f**) was isolated as a white solid (Mp.: 78–79 °C) by silica-gel column chromatography using chloroform as an eluent; ^1^H NMR (500 MHz, CDCl_3_) δ 7.22 (d, *J* = 9.0 Hz, 4H), 6.81 (d, *J* = 9.0 Hz, 4H), 3.93 (t, *J* = 6.4 Hz, 4H), 1.80 (p, *J* = 6.3 Hz, 4H), 1.52 (dd, *J* = 7.2, 3.8 Hz, 4H); ^13^C NMR (500 MHz, CDCl_3_) δ 157.65, 129.26, 125.34, 115.71, 68.08, 29.10, 25.80; HRMS (ESI) calcd for C_24_H_50_: 338.0840, found: 338.0840.

1,6-Di(4-pinacolborylphenyloxy)hexane (**2g**) [49] was isolated as a white solid (Mp.: 139–140 °C) by silica-gel column chromatography using chloroform as an eluent; ^1^H NMR (500 MHz, CDCl_3_) δ 7.75 (d, *J* = 8.56 Hz, 4H), 6.89 (d, *J* = 8.60 Hz, 4H), 3.99 (t, *J* = 7.5 Hz, 4H), 1.82 (t, *J* = 6.25 Hz, 4H) 1.54 (quin, *J* = 3.75 Hz, 4H), 1.34 (s, 24H); ^13^C NMR (500 MHz, CDCl_3_) δ161.68, 136.50, 113.85, 83.51, 67.58, 29.15, 25.85, 24.86.

Diethyl dodecanedioate (**2h**) was isolated as a colorless oil by silica-gel column chromatography using a mixture of hexane and EtOAc (3:1) as an eluent; ^1^H NMR (500 MHz, CDCl_3_) δ 4.12 (t, *J* = 7.1 Hz, 4H), 2.32–2.25 (m, 4H), 1.66–1.54 (m, 8H), 1.33–1.21 (m, 14H); ^13^C NMR (500 MHz, CDCl_3_) δ 173.93, 60.14, 60.06, 34.37, 29.22, 29.11, 24.94, 14.22; HRMS (ESI) calcd for C_16_H_31_O_4_ [M+H]^+^: 287.2222, found: 287.2221.

1,6-Diphthalimidylhexane (**2i**) [22] was isolated as a white solid (Mp.: 180–181 °C) by silica-gel column chromatography using a mixture of hexane and EtOAc (3:1) as an eluent; ^1^H NMR (500 MHz, CDCl_3_) δ 7.83 (dd, *J* = 5.4, 3.0 Hz, 4H), 7.70 (dd, *J* = 5.5, 3.0 Hz, 4H), 3.67 (t, *J* = 7.3 Hz, 4H), 1.67 (quint, *J* = 7.1 Hz, 4H), 1.38 (quint, *J* = 3.5 Hz, 4H); ^13^C NMR (500 MHz, CDCl_3_) δ 168.42, 133.83, 132.14, 123.16, 37.87, 28.44, 26.41.

1,6-Di(tert-butyldimethylsilyloxy)hexane (**2j**) was isolated as a colorless oil by silica-gel column chromatography using a mixture of hexane and EtOAc (10:1) as an eluent; ^1^H NMR (400 MHz, CDCl_3_) δ 3.60 (t, *J* = 6.6 Hz, 4H), 1.51 (q, *J* = 6.7 Hz, 4H), 1.32 (ddd, *J* = 7.3, 4.5, 3.3 Hz, 4H), 0.89 (s, 18H), 0.04 (s, 12H); ^13^C NMR (126 MHz, CDCl_3_) δ 63.24, 32.86, 25.98, 25.61, 18.37, −5.27; HRMS (ESI) calcd for C_18_H_43_O_2_Si_2_ [M+H]^+^: 347.2802, found: 347.2802.

2,7-Di(tert-butyldimethylsilyloxy)octane (**2k**) was isolated as a 1:1 mixture of *dl*- and *meso*-form (colorless oil) by silica-gel column chromatography using a mixture of hexane and EtOAc (10:1) as an eluent; ^1^H NMR (500 MHz, CDCl_3_) δ 3.76 (dq, *J* = 11.8, 6.0 Hz, 2H), 1.49–1.18 (m, 8H), 1.11 (d, *J* = 6.1 Hz, 6H), 0.88 (s, 18H), 0.042 and 0.040 (s, 12H); all signals were obscured except for the peaks at 0.04 ppm; ^13^C NMR (126 MHz, CDCl_3_) δ 68.61, 39.76, 25.91, 25.87, 23.80, 18.17, −4.72, assignable signals for another isomer: 68.57, 25.85, 23.82, 18.17, −4.42; HRMS (ESI) calcd for C_20_H_47_O_2_Si_2_ [M+H]^+^: 375.3115, found: 375.3109.

7,10-Dibutylhexadecane (**2l**) was isolated as a 1:1 mixture of *dl*- and *meso*-form (colorless oil) by silica-gel column chromatography using hexane as an eluent; ^1^H NMR (500 MHz, CDCl_3_) δ 1.34–1.16 (m, 38H), 0.93–0.84 (m, 12H); ^13^C NMR (126 MHz, CDCl_3_) δ 37.71, 33.69, 33.39, 31.98, 30.28, 29.84, 29.00, 26.69, 23.18, 22.72, 14.19, 14.13; HRMS (ESI) calcd for C_24_H_50_: 338.3913, found: 338.3906.

1,2-Bis(*trans*-4-butylcyclohexyl)ethane (**2m**) was isolated as a white solid (Mp.: 89–90 °C) by silica-gel column chromatography using hexane as an eluent; ^1^H NMR (500 MHz, CDCl_3_) δ 1.76–1.67 (m, 8H), 1.32–1.20 (m, 8H), 1.19–1.10 (m, 12H), 0.91–0.80 (m, 14H).; ^13^C NMR (126 MHz, CDCl_3_) δ 38.20, 37.87, 37.23, 34.83, 33.43, 33.40, 29.26, 23.03, 14.16; HRMS (ESI) calcd for C_22_H_42_: 306.3287, found: 306.3290.

## 4. Conclusions

In summary, we have established a direct homo-coupling of alkyl tosylates using a combination of nickel and the nucleophilic cobalt-hybrid catalyst system in the presence of an Mn reductant. A diverse set of functional groups on alkyl tosylates can be tolerated in the homo-coupling, giving rise to the corresponding alkyl dimers in good yields under mild conditions. Although the homo-coupling was sensitive to the bulkiness of alkyl tosylates, a longer reaction time gave the corresponding homodimer. Mechanistic studies using a MeCbl catalyst strongly suggested a formation of the alkyl-Co(III) intermediate in the homo-coupling. Moreover, the addition of the hydrogen-atom donor, γ-terpinene, into the reaction revealed a generation of alkyl radicals during the reaction. Further mechanistic studies and synthetic applications of this Ni/Co-hybrid catalyst system are underway in our laboratory.

## Figures and Tables

**Table 1:**
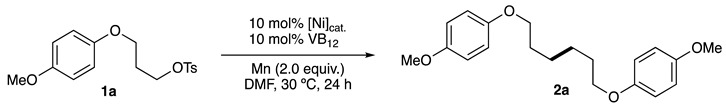
The screening of the reaction conditions in the homo-coupling of **1a**.

Entry	Ni catalysts	Yield of 2a (%) ^a^	Conversion of 1a (%) ^a^
1	NibpyCl_2_	72	100
2 ^b^	NibpyCl_2_	0	20
3	None	0	32
4 ^c^	NibpyCl_2_	0	0
5 ^d^	NibpyCl_2_	38	65
6	Ni(4,4’-(MeO_2_C)_2_bpy)Cl_2_	38	76
7	Ni(4,4’-*^t^*Bu_2_bpy)Cl_2_	18	67
8	Ni(4,4’-Mes_2_bpy)Cl_2_^e^	38	78
9	Ni[4,4’-(MeO)_2_bpy]Cl_2_	10	66
10	Ni(6-Mebpy)Cl_2_	42	92
11	Ni(6,6’-Me_2_bpy)Cl_2_	17	95
12	Ni(1,10-phen)Cl_2_	75	100
13	Ni(4,7-Ph_2_phen)Cl_2_	60	100
14 ^f^	NiphenCl_2_	82	100
15 ^g^	NiphenCl_2_	0	0
16 ^f^	NiphenBr_2_	93	100
17 ^h^	NiphenBr_2_	21–28	51–59

^a^ Determined by GC. ^b^ Without VB_12_. ^c^ Without Mn. ^d^ Zn instead of Mn. ^e^ Mes = 2,4,6-Trimethylphenyl. ^f^ DMSO was used instead of DMF. ^g^ THF, 1,4-dioxane, or acetonitrile were used instead of DMF. ^h^ CoCl(dmg)_2_L (dmg = dimethylglyoximato, L = pyridine derivatives) were used instead of VB_12_.

**Table 2:**
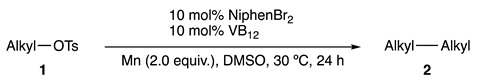
The substrate scope in the Ni/Co-catalyzed homo-coupling.

Entry	Alkyl Tosylates 1		Product 2 and Yield (%) ^a^		
1	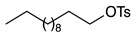	**1b**	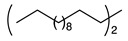	**2b**	86
2	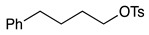	**1c**	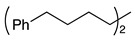	**2c**	70
3	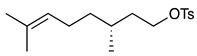	**1d**	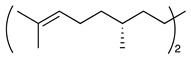	**2d**	67
4	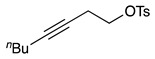	**1e**	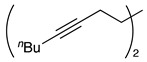	**2e**	63
5	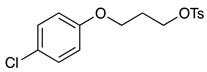	**1f**	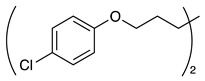	**2f**	65
6	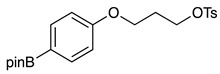	**1g**	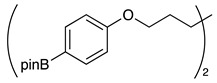	**2g**	75
7	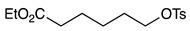	**1h**	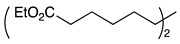	**2h**	70
8 ^b^	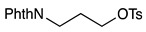	**1i**	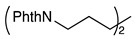	**2i**	60
9	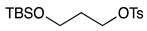	**1j**	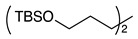	**2j**	73
10	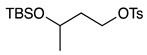	**1k**	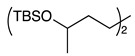	**2k**	90 [1:1] ^c^
11 ^d,e^	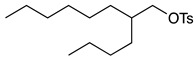	**1l**	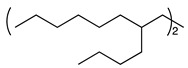	**2l**	80 [1:1] ^c^
12 ^e, f^	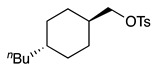	**1m**	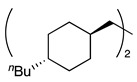	**2m**	65

^a^ Isolated yields. ^b^ PhthN = Phthalimidyl. ^c^ The bracket value indicates a ratio of the *dl*- and *memo*-dimers estimated by NMR spectra. ^d^ Reaction times: 48 h. ^e^ DMF was used instead of DMSO. ^f^ Reaction times: 74 h.

## Supplementary Materials

The ^1^H and ^13^C NMR spectra of homo-coupling products are available online.
