# Supplementary material for: Ni/Co-Catalyzed Homo-Coupling of Alkyl Tosylates
Source: Molecules. 2019 Apr 12;24(8):1458. doi: 10.3390/molecules24081458 (PMC6515247; doi:10.3390/molecules24081458)

*Supplementary Materials for*

# Ni/Co-Catalyzed Homo-Coupling of Alkyl Tosylates

Kimihiro Komeyama *, Ryusuke Tsunemitsu, Takuya Michiyuki, Hiroto Yoshida and Itaru Osaka

*Department of Applied Chemistry, Graduate School of Engineering, Hiroshima University*
*1-4-1 Kagamiyama, Higashi-Hiroshima City, Hiroshima 739-8527, Japan*
**Correspondence: kkome@hiroshima-u.ac.jp*

1. A GC chart after work-up (entry 1, Table 1)
2. $^{1}$H and $^{13}$C NMR spectra of homo-coupling products

1. A GC chart after work-up (entry 1, Table 1)

File No.:25-quench+IS.gcd (Method:Normal30min.gcm) Date:2018/06/08 10:29:31

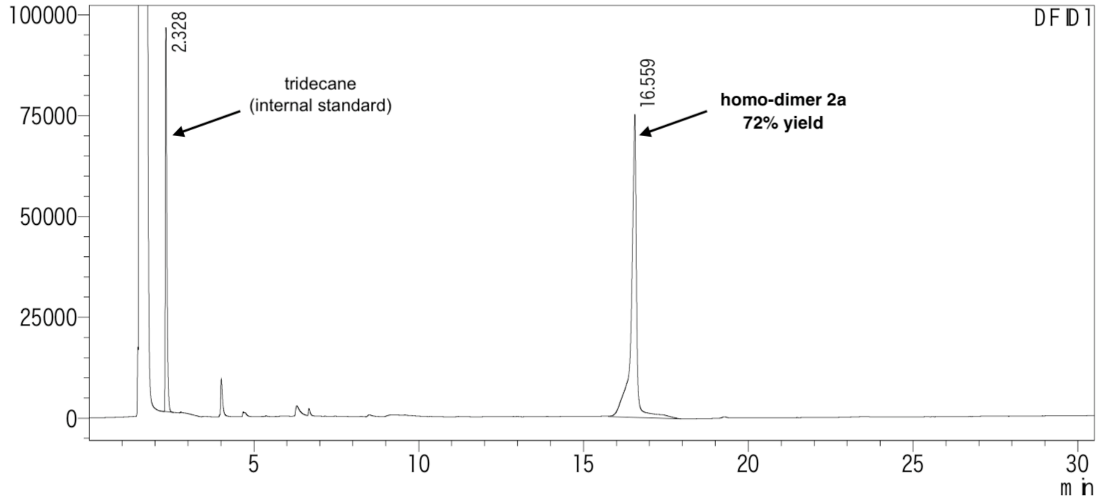


DFID1

| ピーク# | 保持時間 | 面積 | 高さ | 濃度 | 単位 | マーク | 化合物名 |
|---|---|---|---|---|---|---|---|
| 1 | 2.328 | 299082 | 94901 | 0.000 | | M | |
| 2 | 16.559 | 836143 | 75056 | 0.000 | | V | |
| 合計 | | 1135225 | 169957 | | | | |

2. 1H and 13C NMR spectra of homo-coupling products

012_1H NMR (CDCl3, 399.89 MHz)

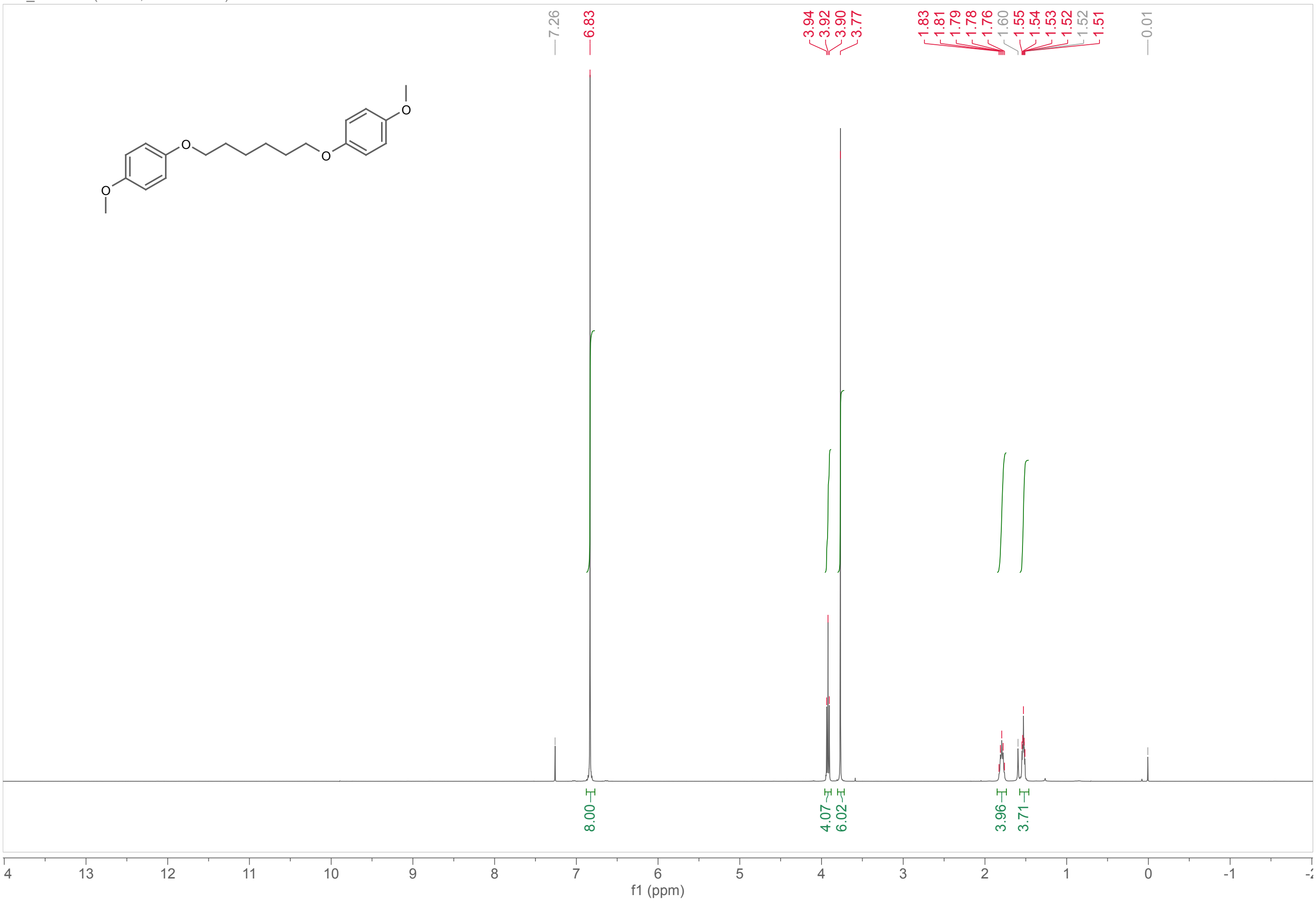


012_13C NMR (CDCl3, 125.72 MHz)

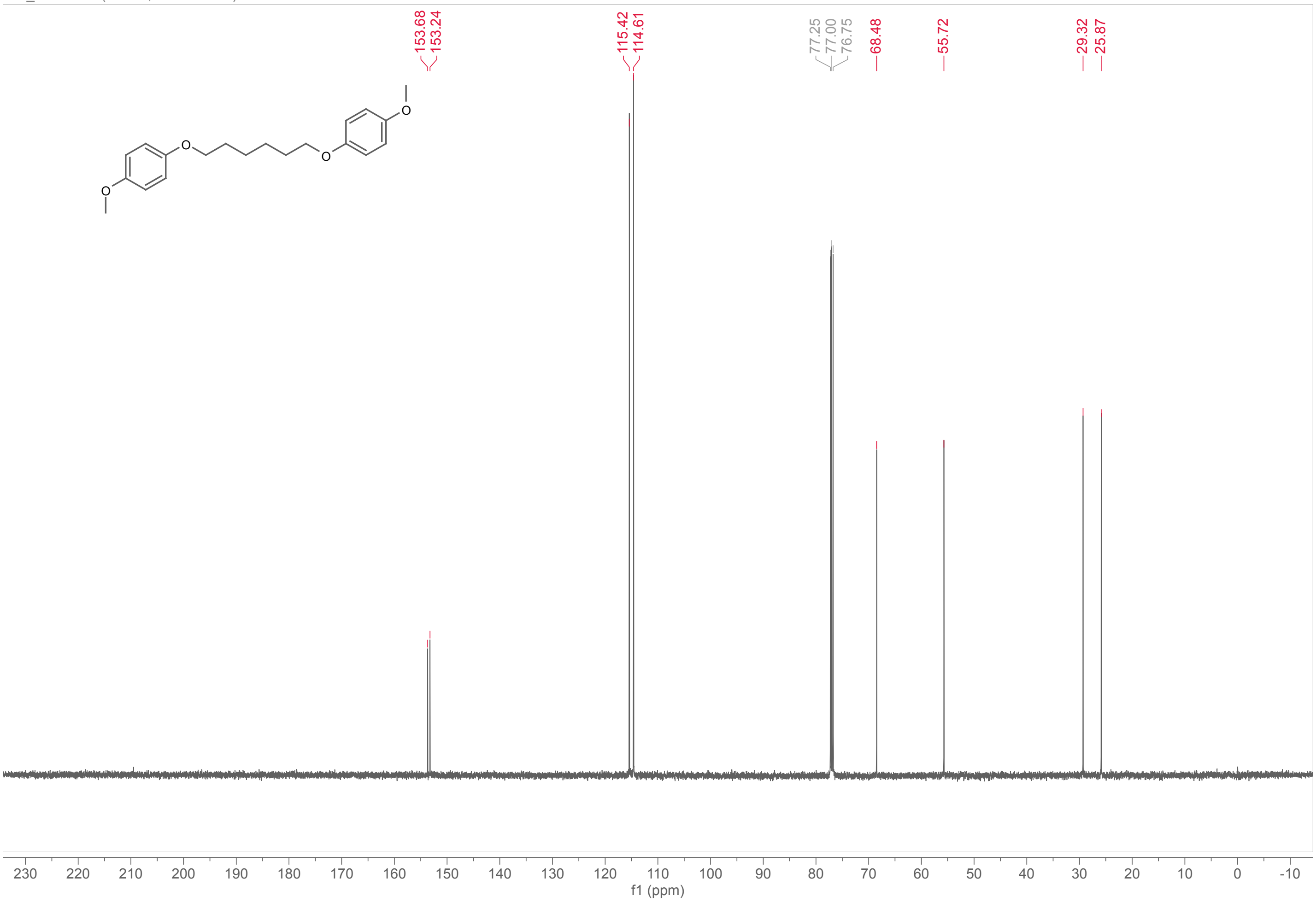

89_1H NMR (CDCl3, 399.89 MHz)

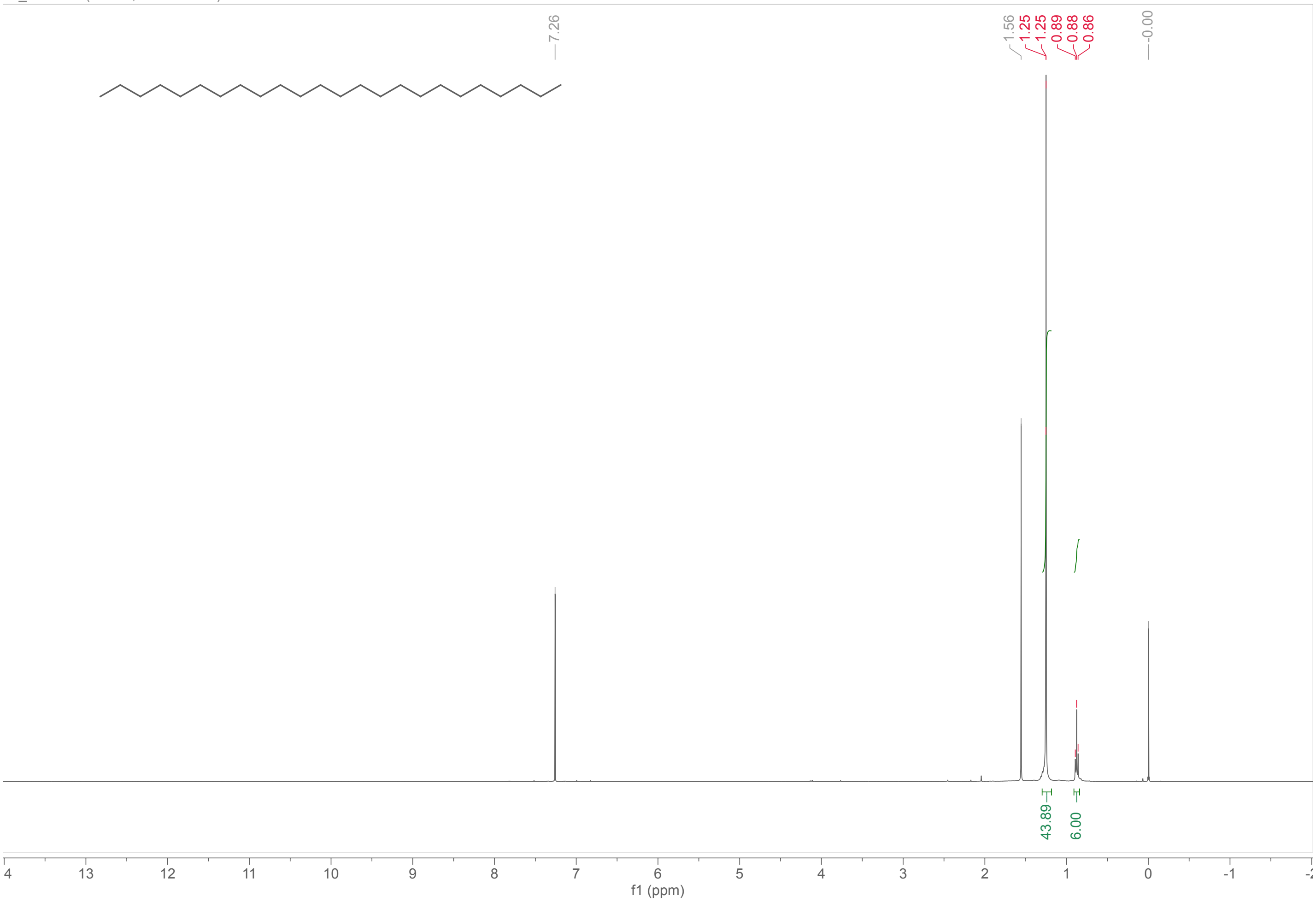


89_13C NMR (CDCl3, 125.72 MHz)

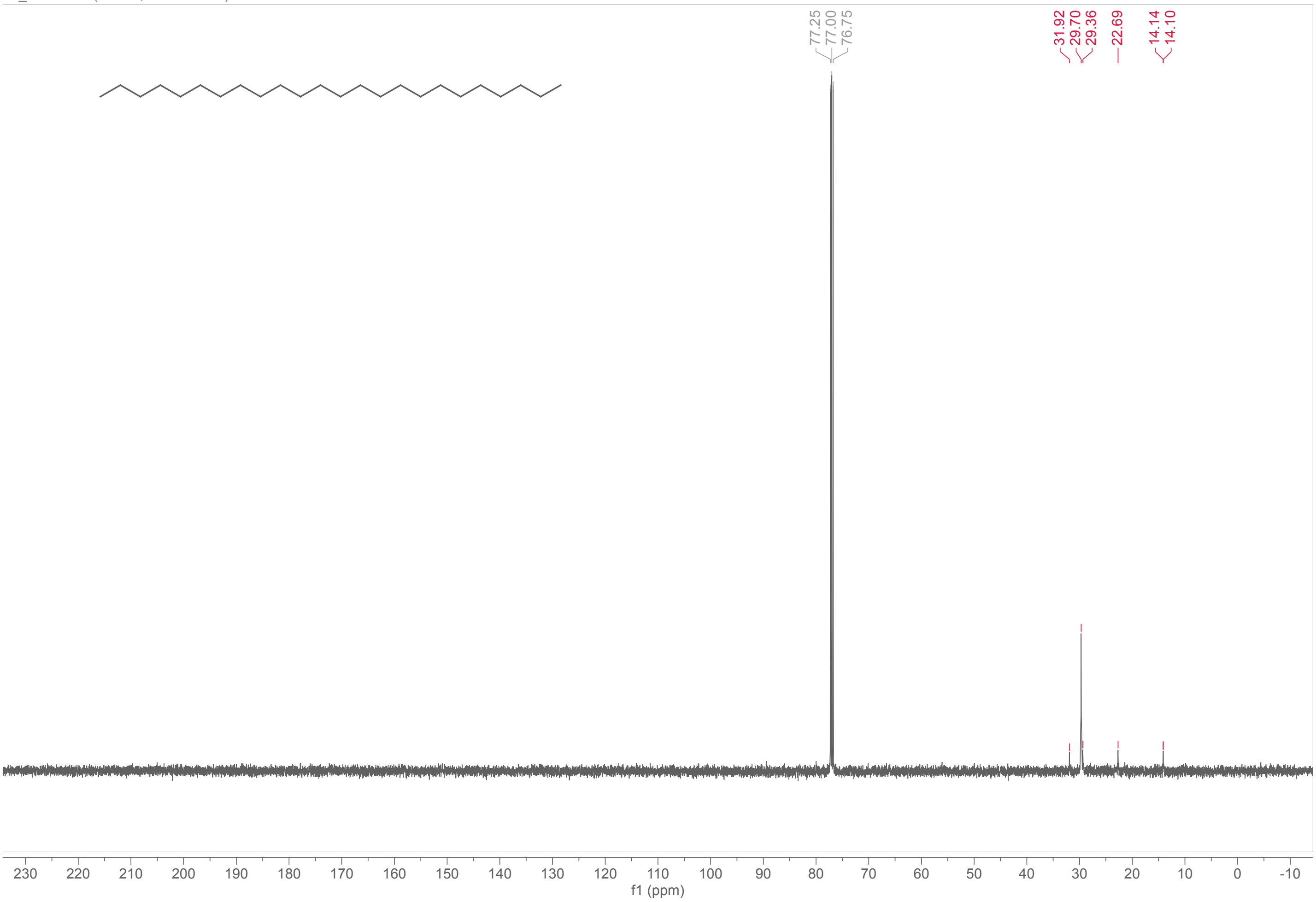

99_1H NMR (CDCl3, 399.89 MHz)

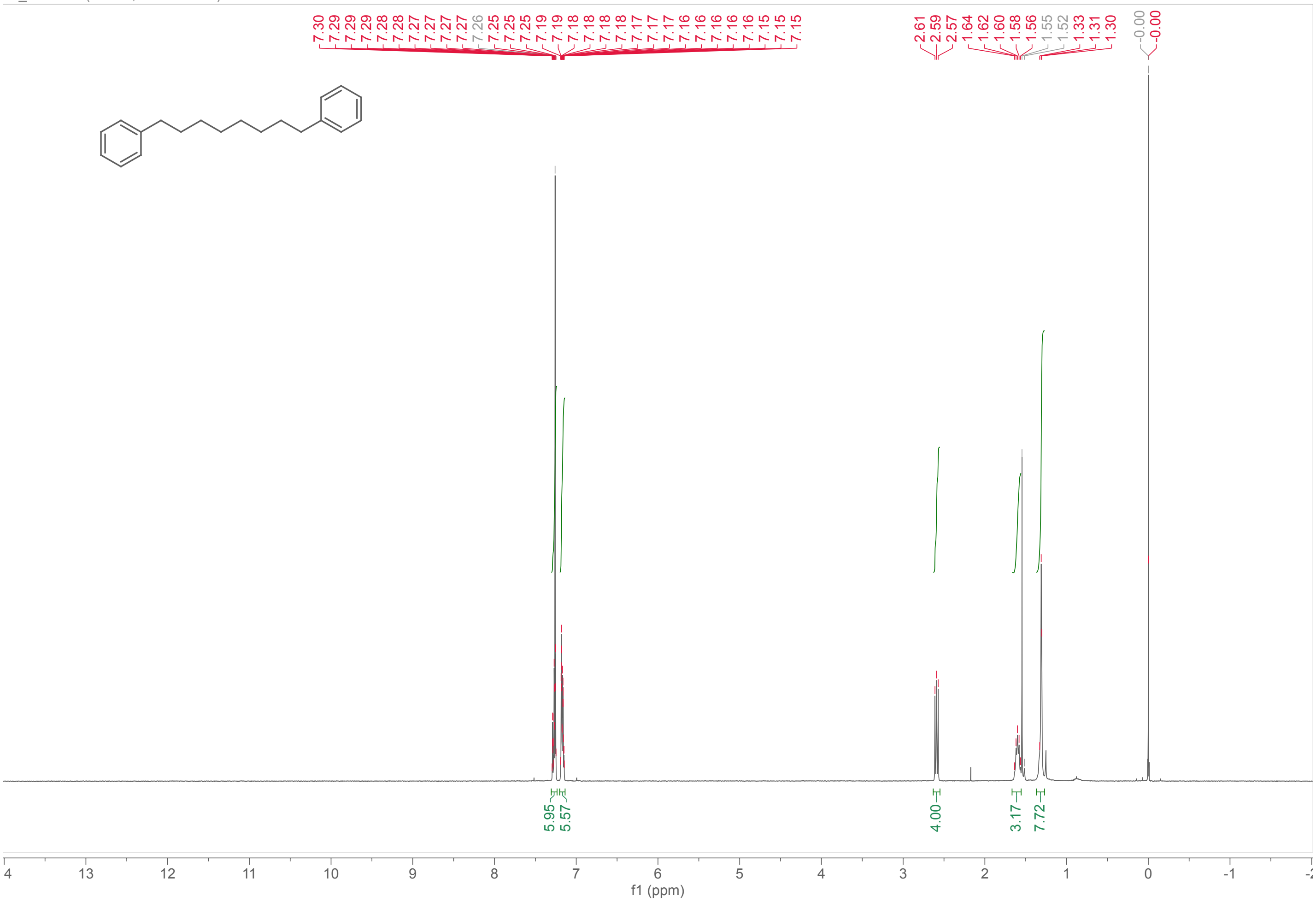


99_13C NMR (cdcl3, 125.72 MHz)

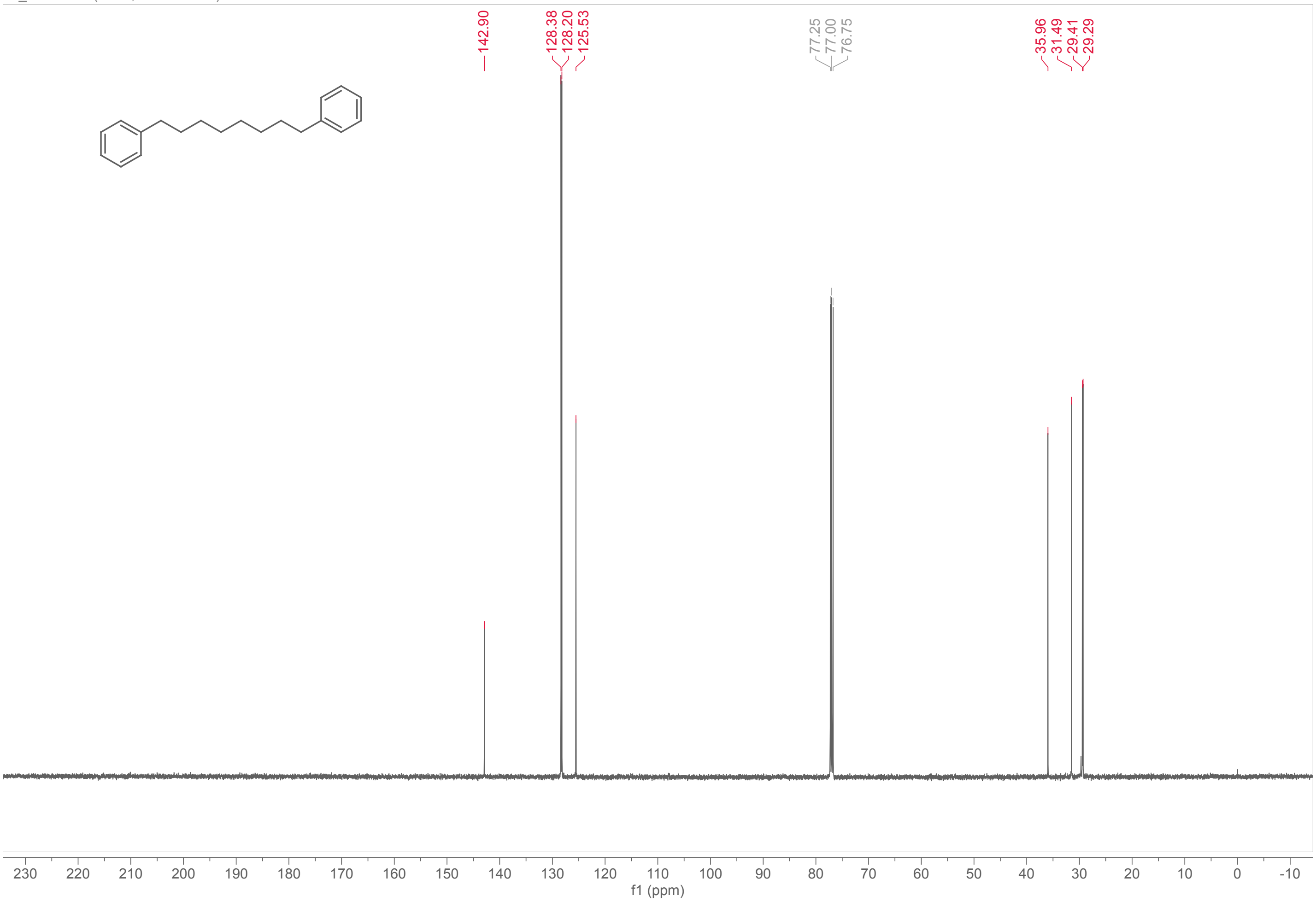

102_1H NMR (CDCl3, 499.94 MHz)

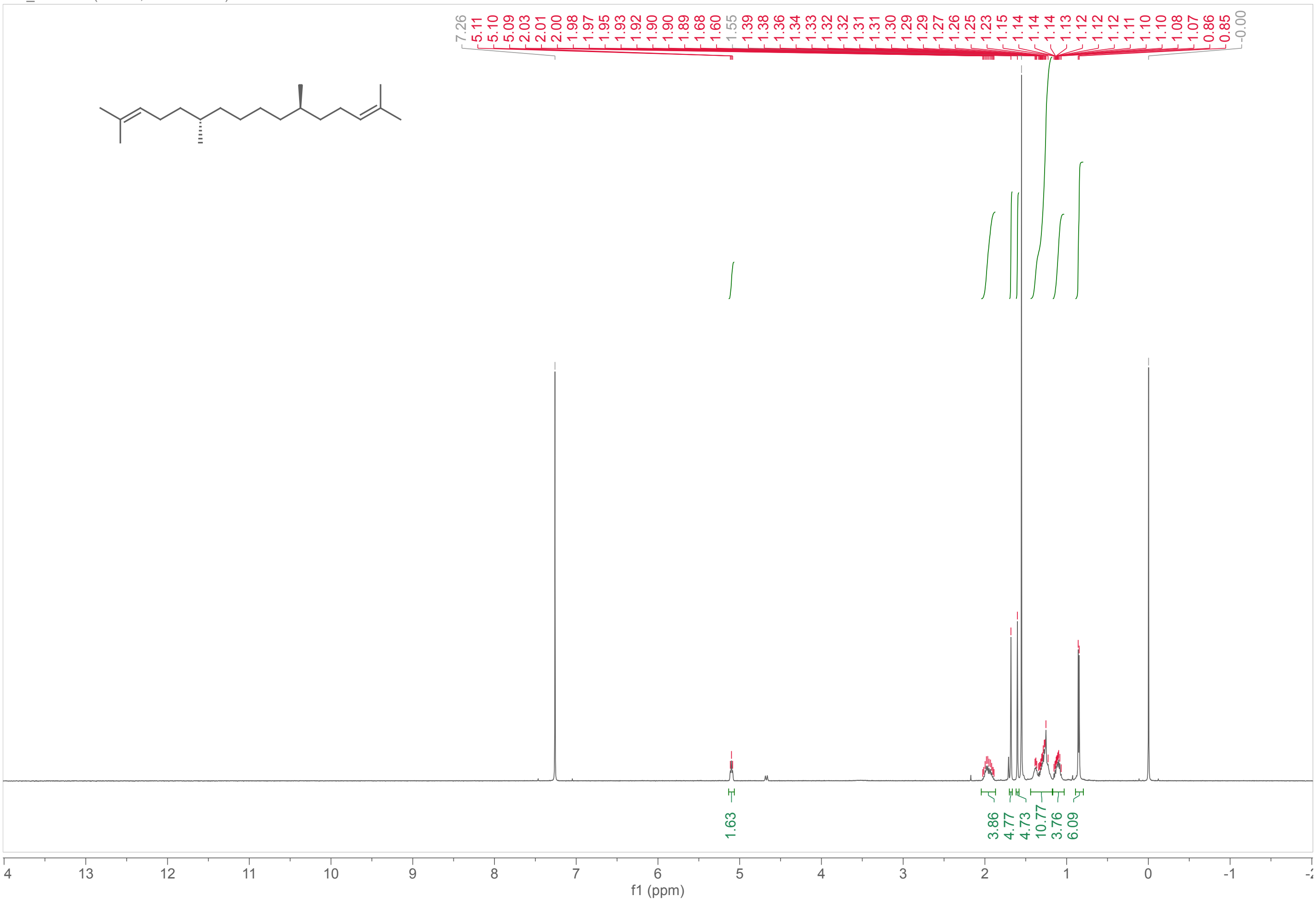


102_13C NMR (CDCl3, 125.72 MHz)

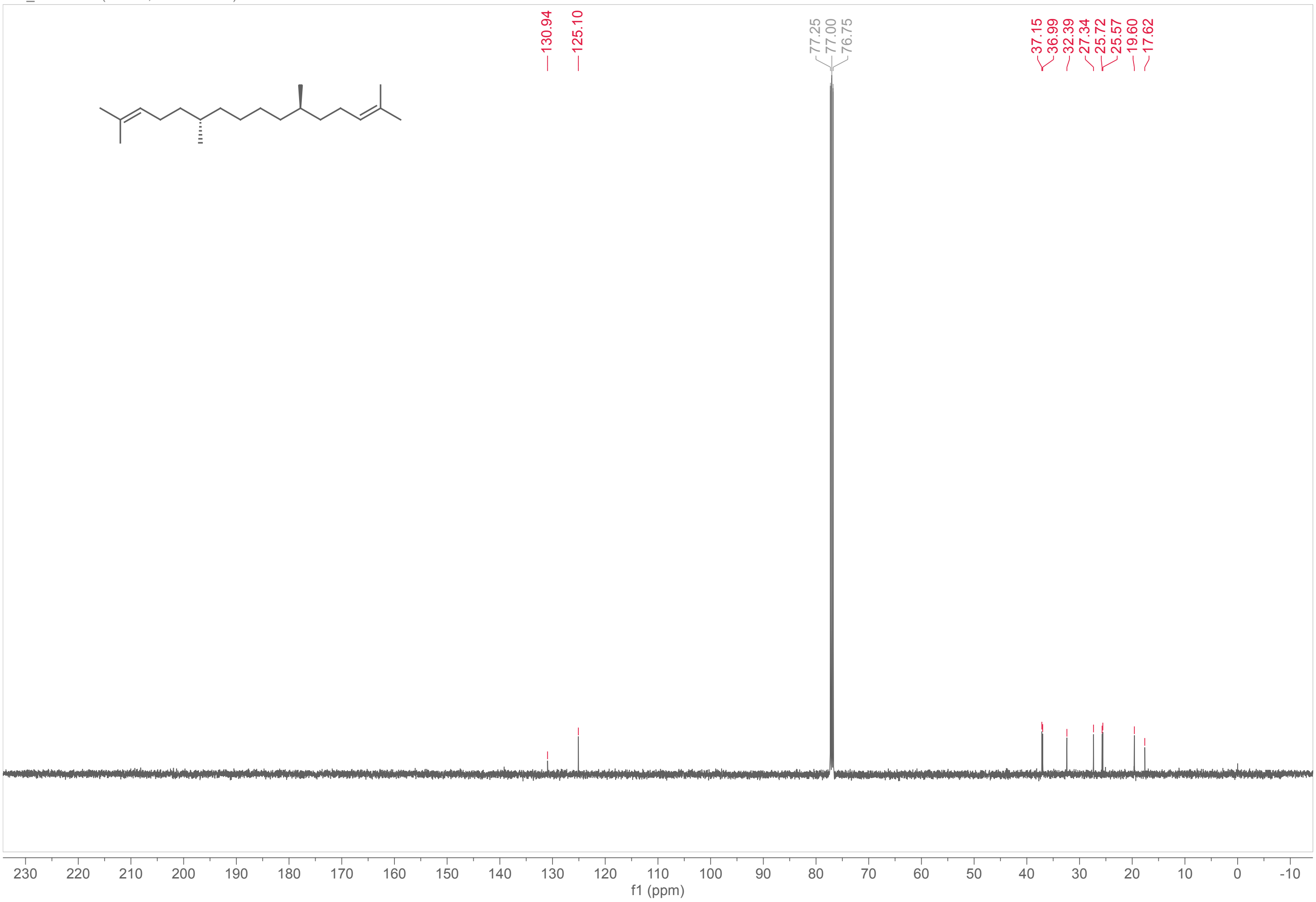

103_1H NMR (CDCl3, 499.94 MHz)

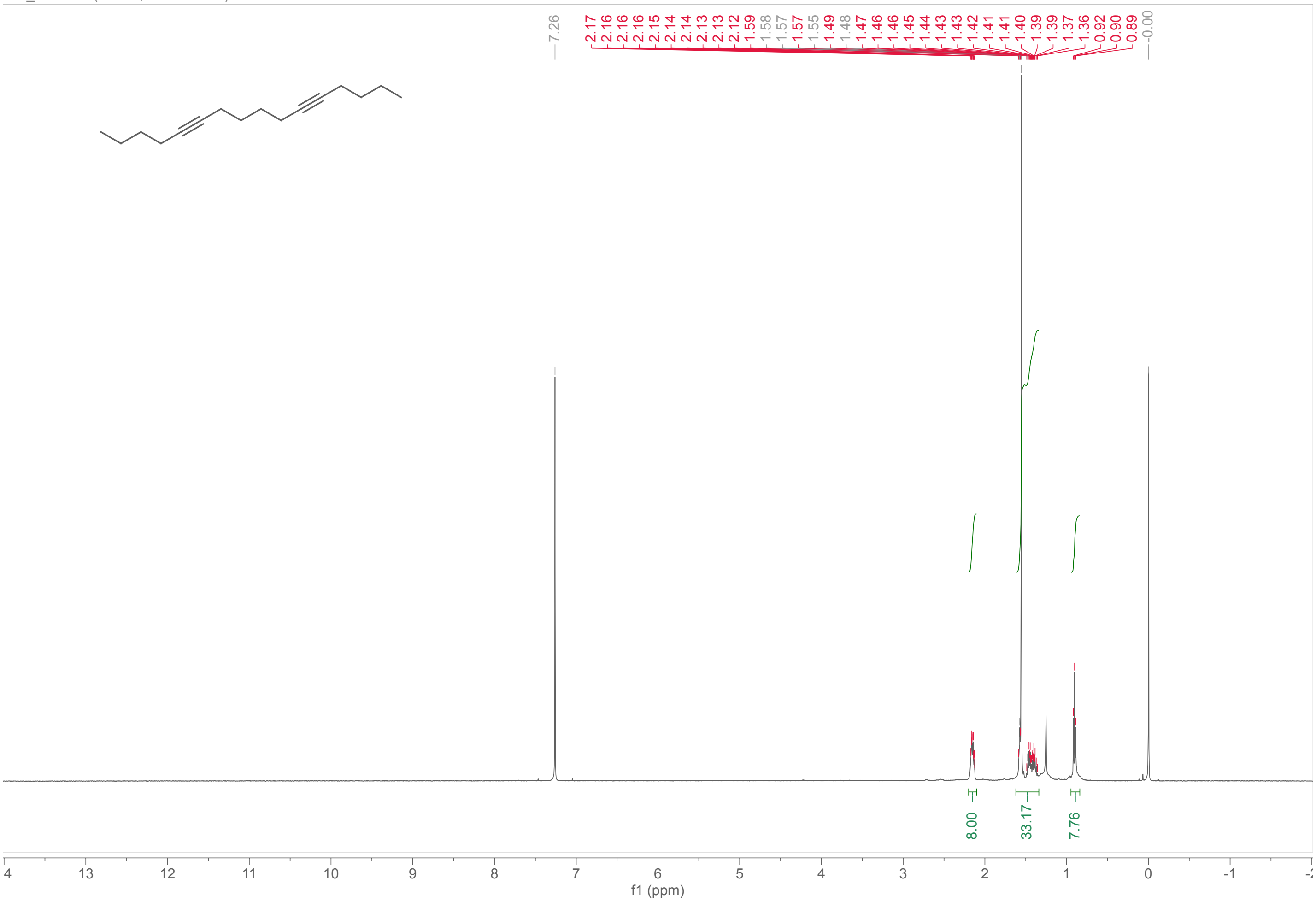


103_13C NMR (CDCl3, 125.72 MHz)

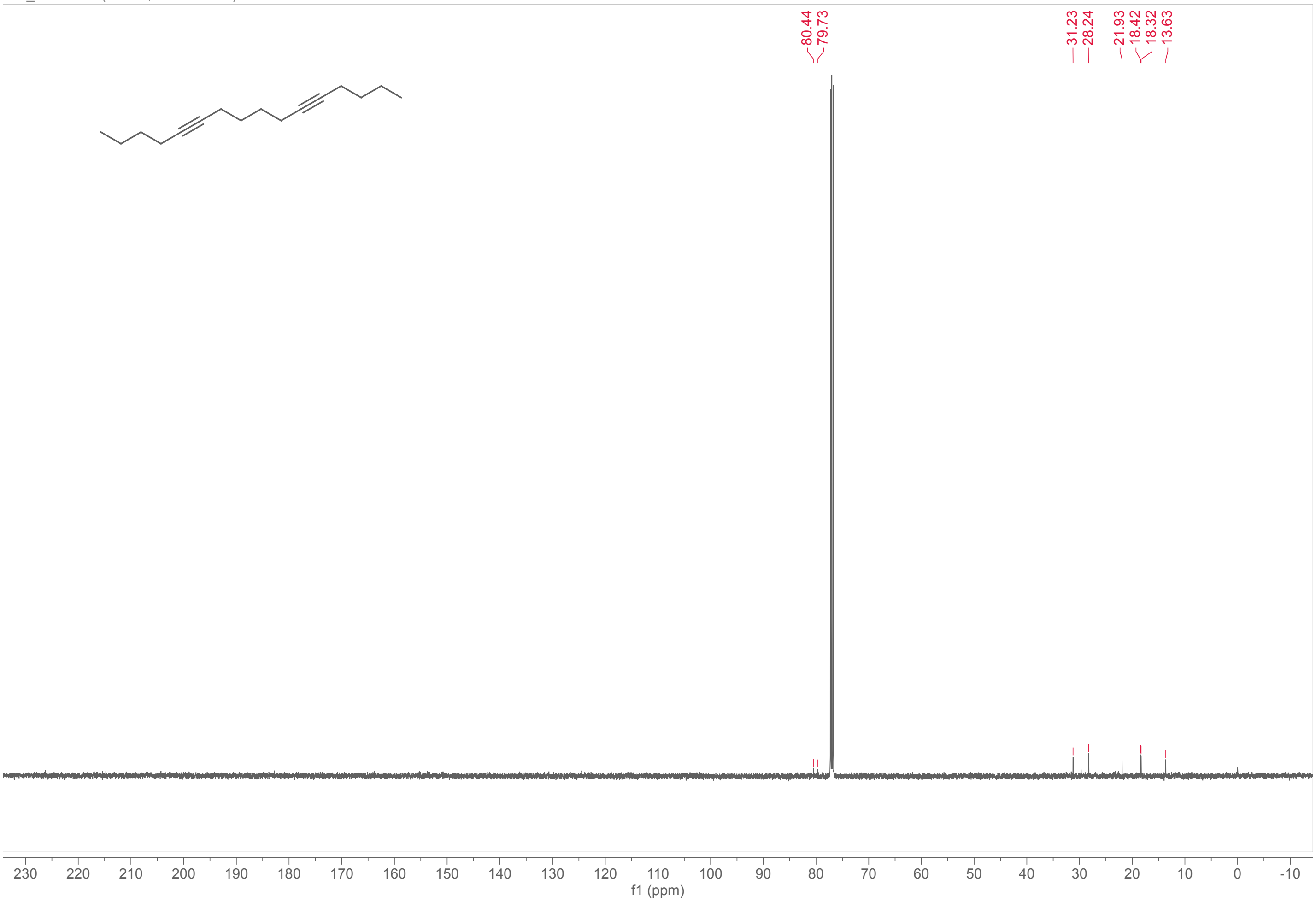

106_1H NMR (CDCl3, 499.94 MHz)

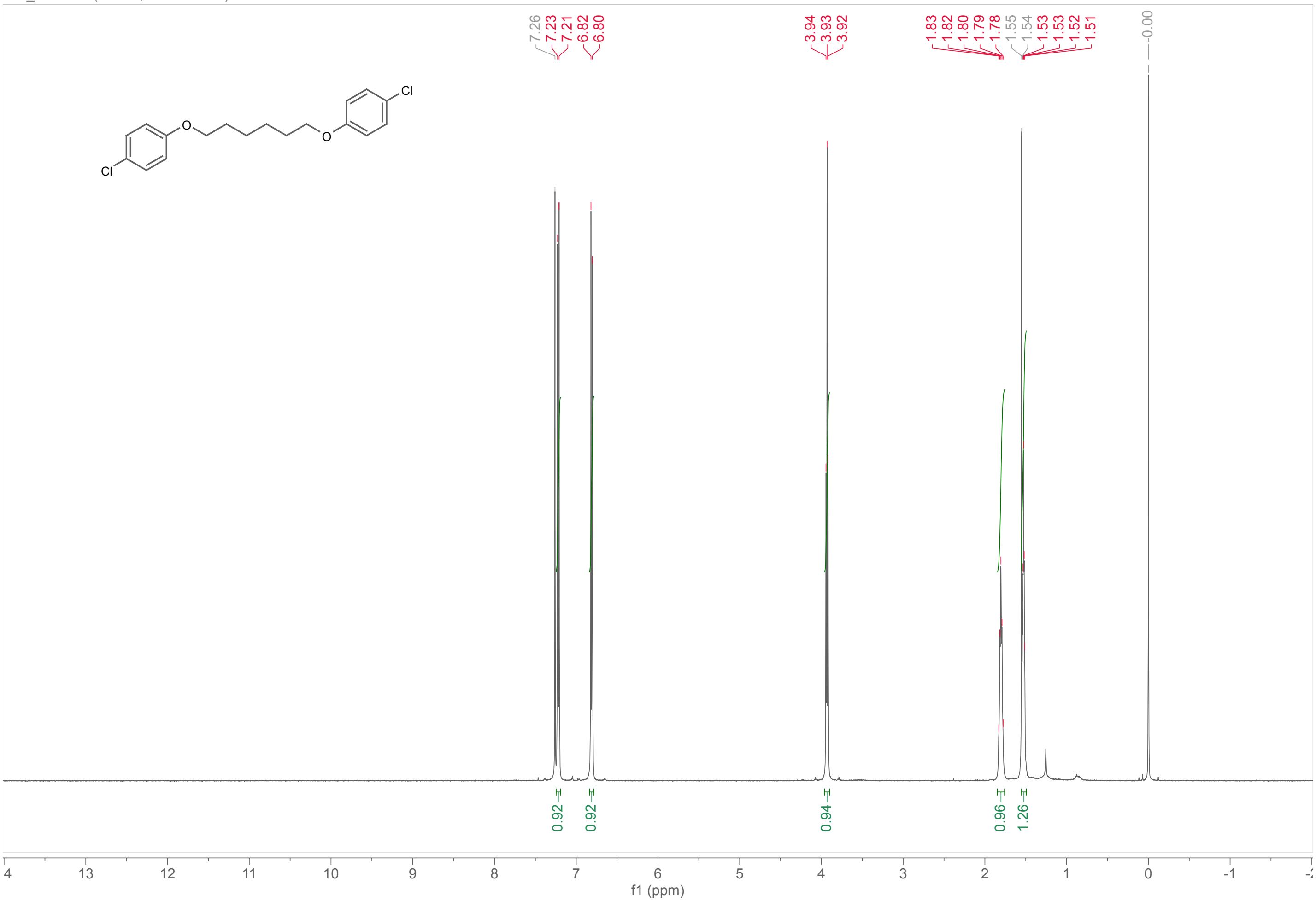


106_13C NMR (CDCl3, 125.72 MHz)

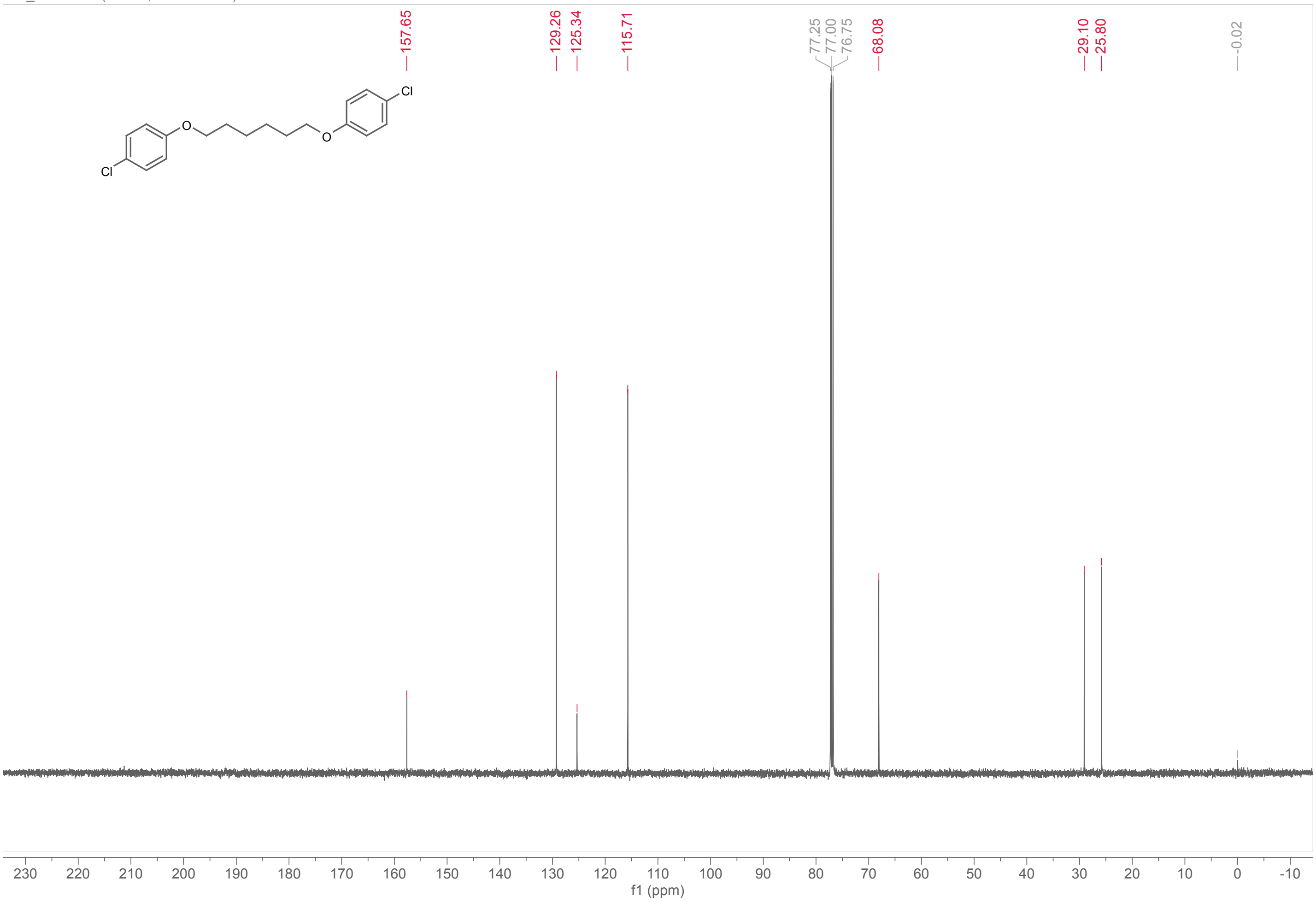

155_1H NMR (CDCl3, 499.94 MHz)

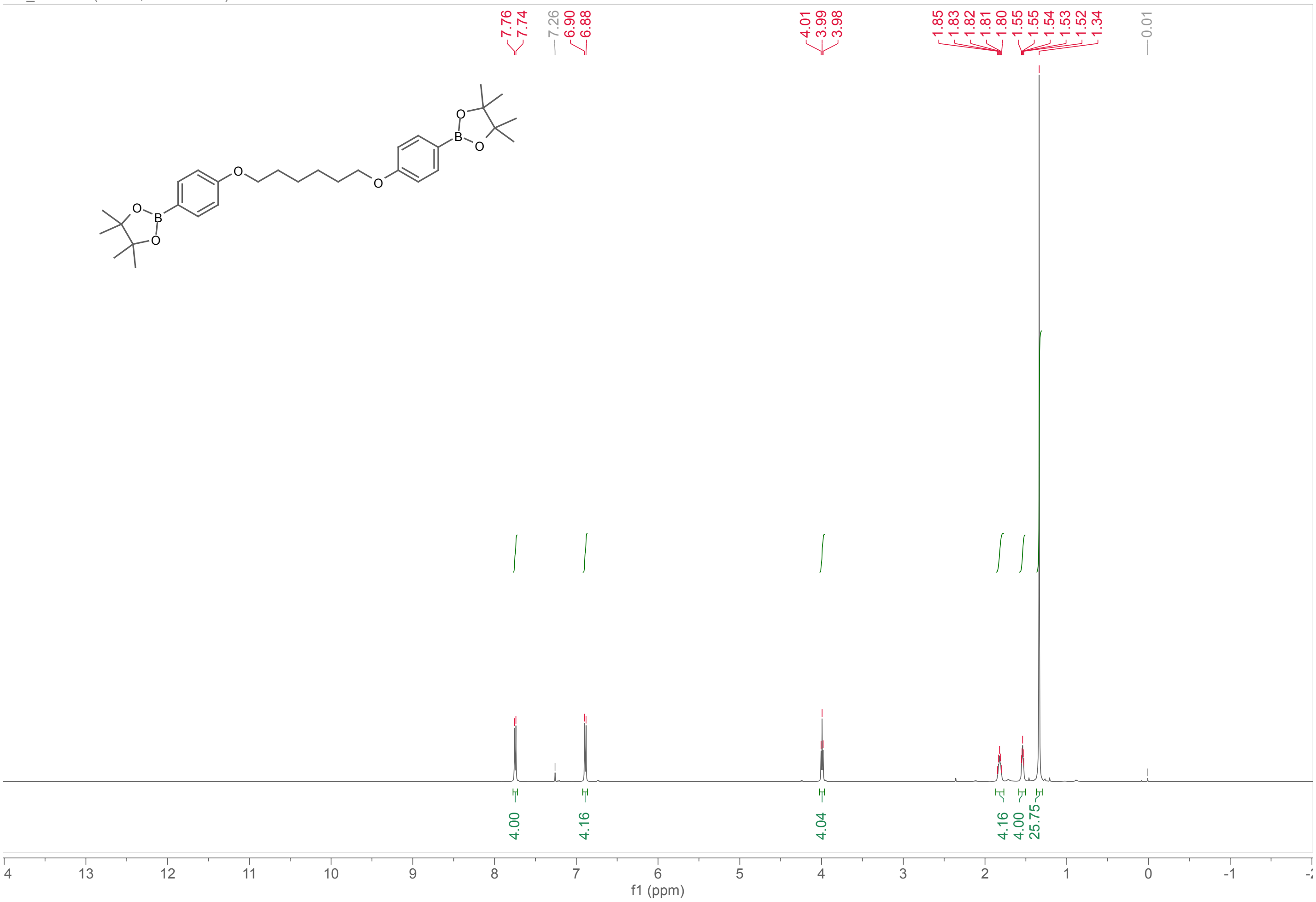


155_13C NMR (CDCl3, 125.72 MHz)

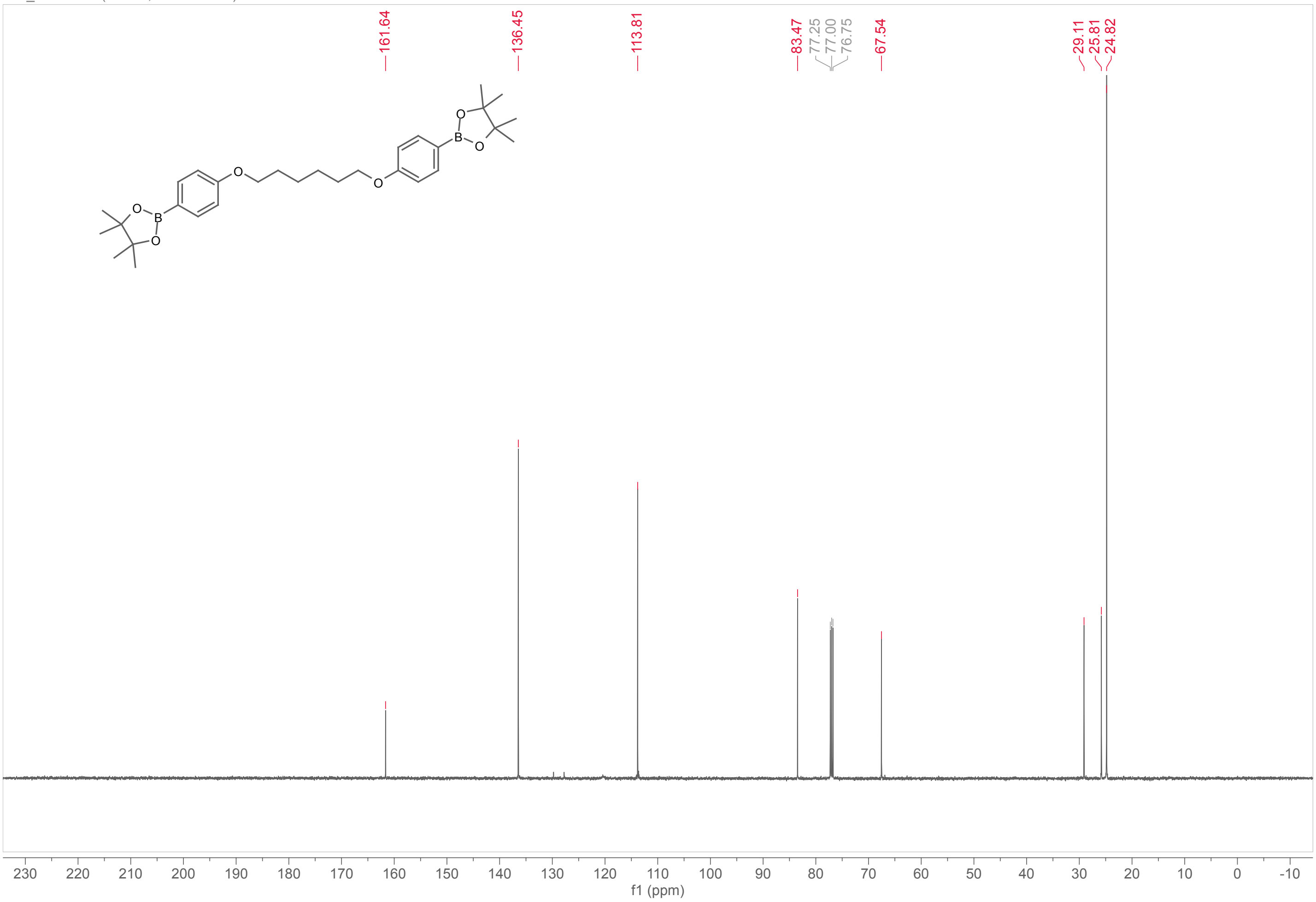

109_1H NMR (CDCl3, 499.94 MHz)

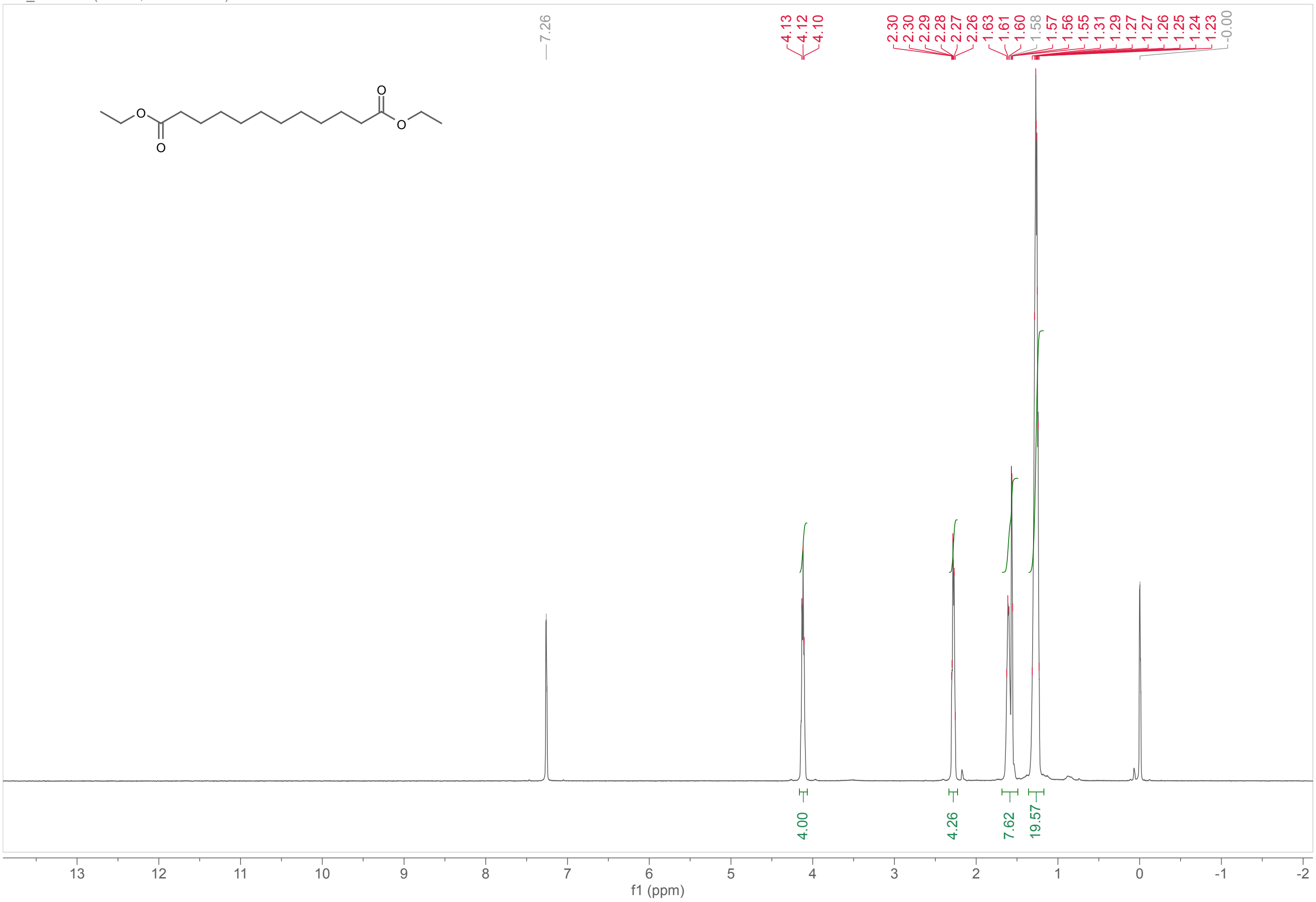


109_13C NMR (CDCl3, 125.72 MHz)

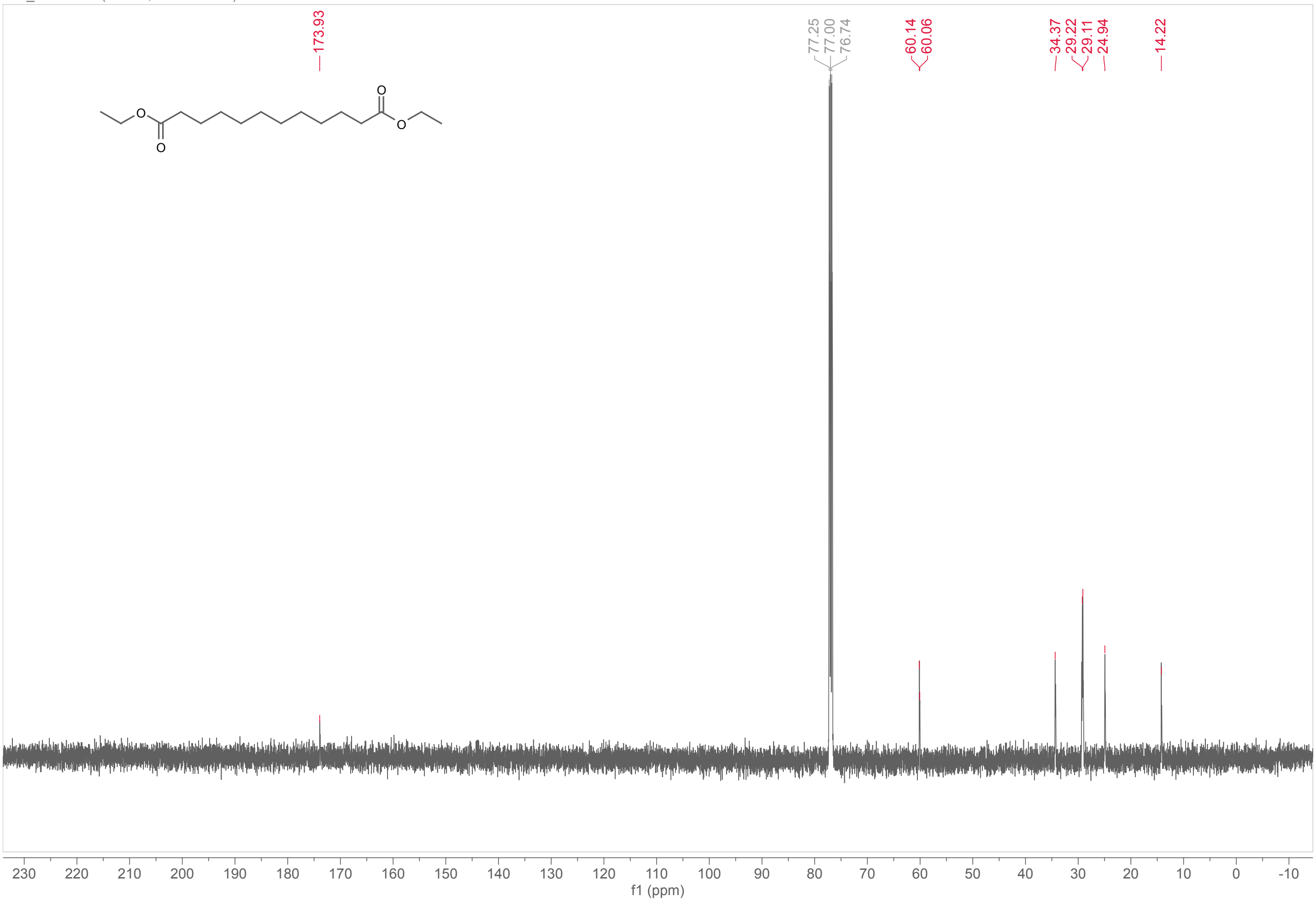

95_1H NMR (CDCl3, 499.94 MHz)

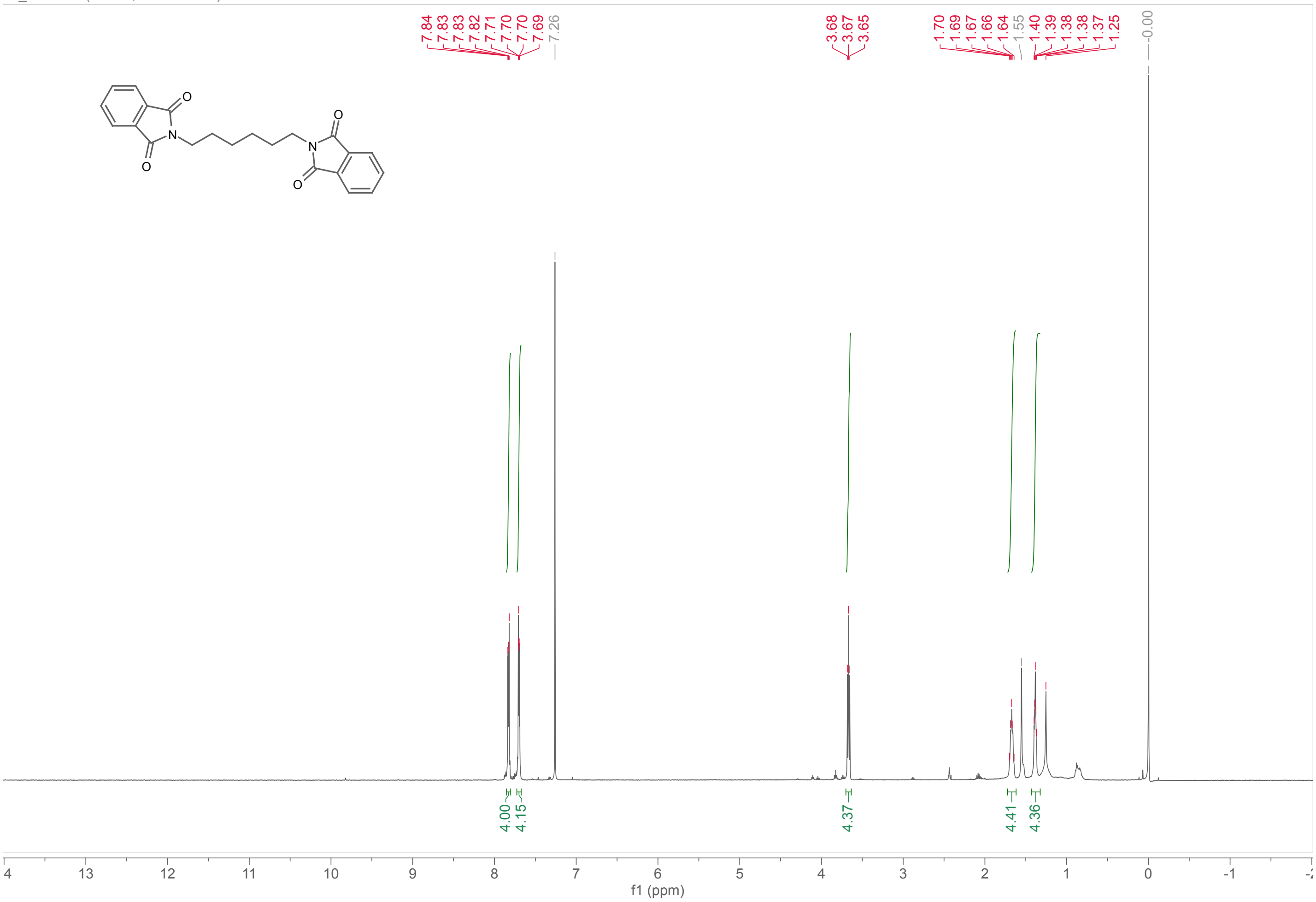


95_13C NMR (CDCl3, 125.72 MHz)

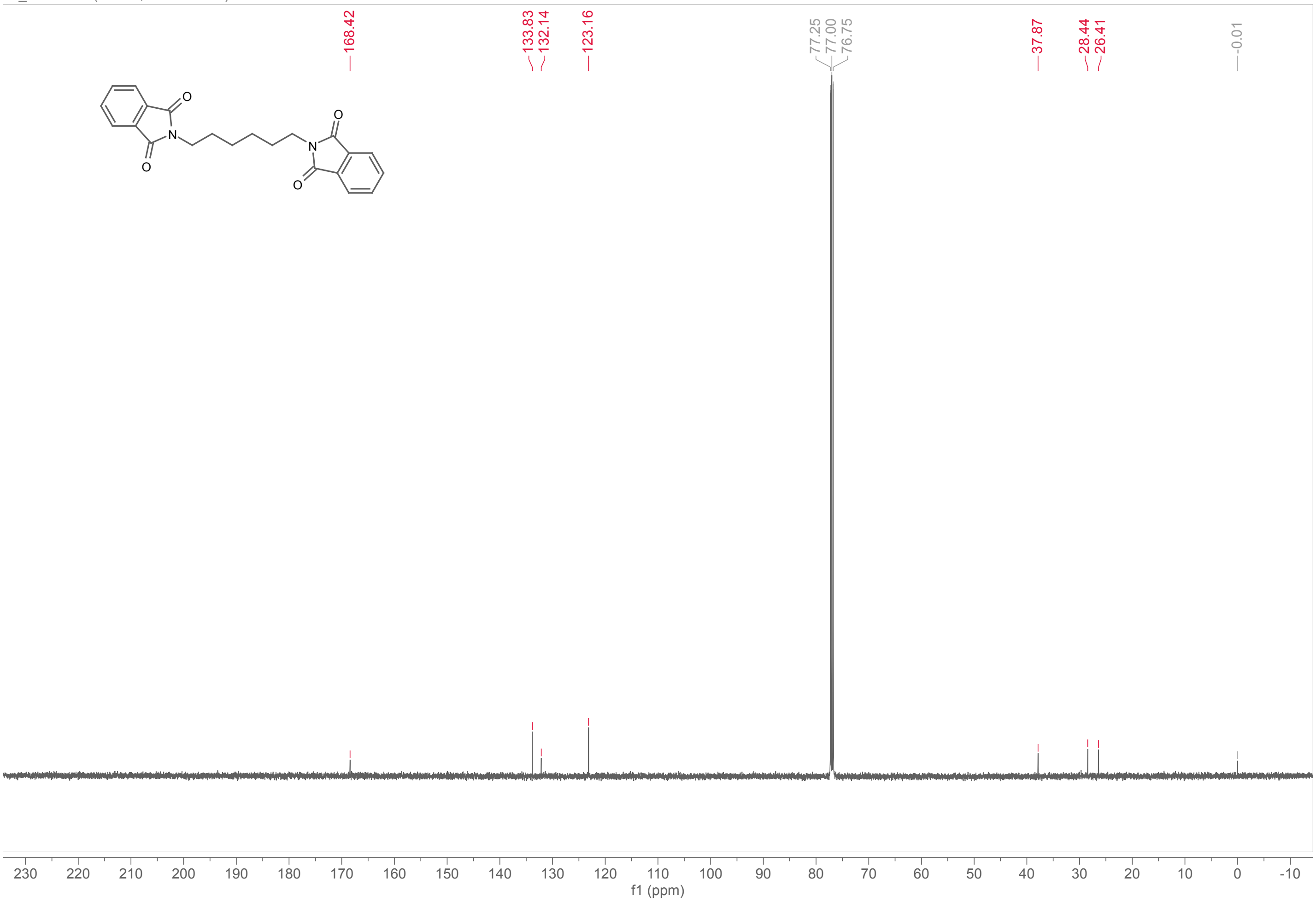

STANDARD PHOSPHORUS PARAMETERS — _1H NMR (CDCl3, 399.89 MHz)

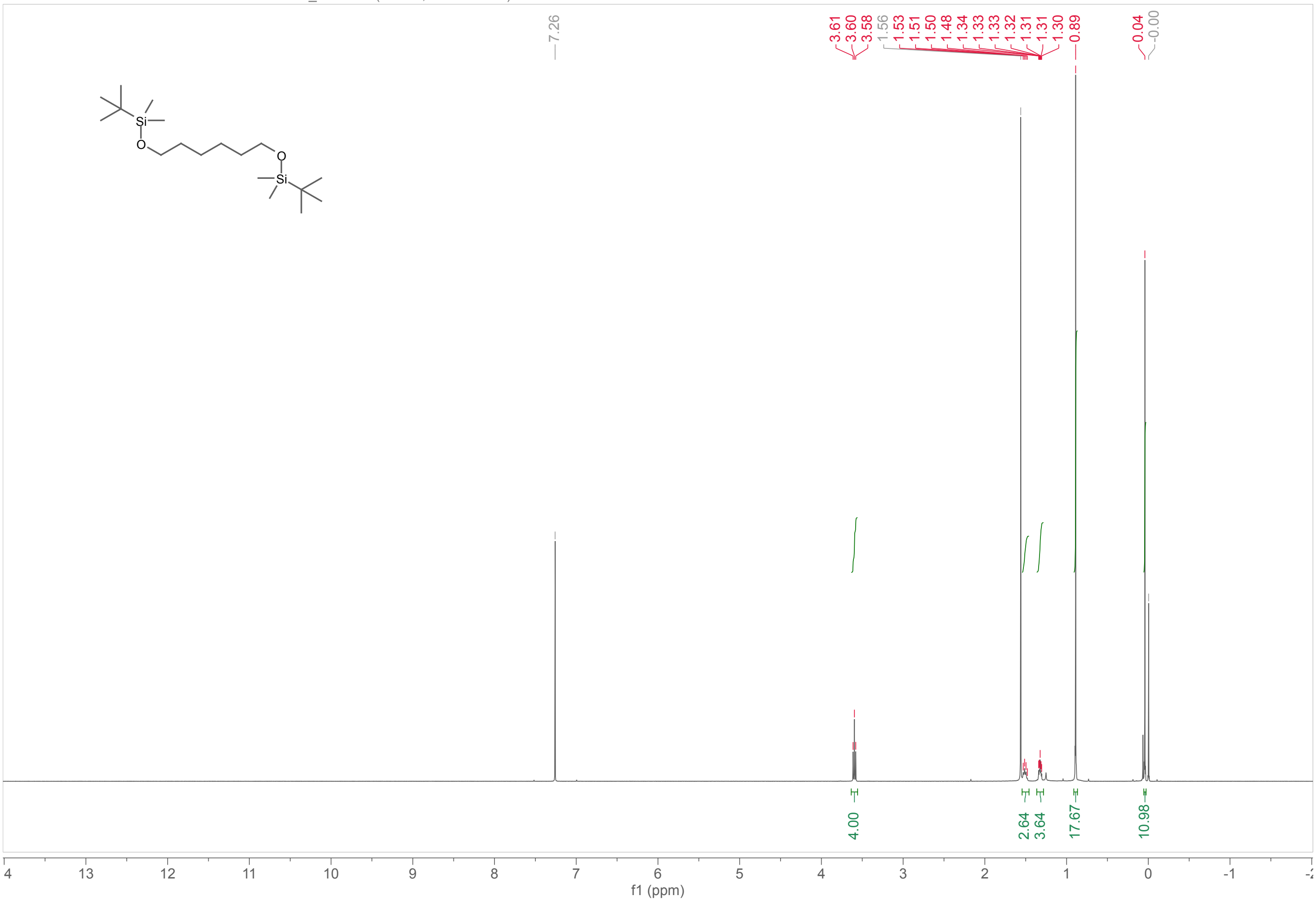

STANDARD PHOSPHORUS PARAMETERS — _13C NMR (CDCl3, 125.72 MHz)

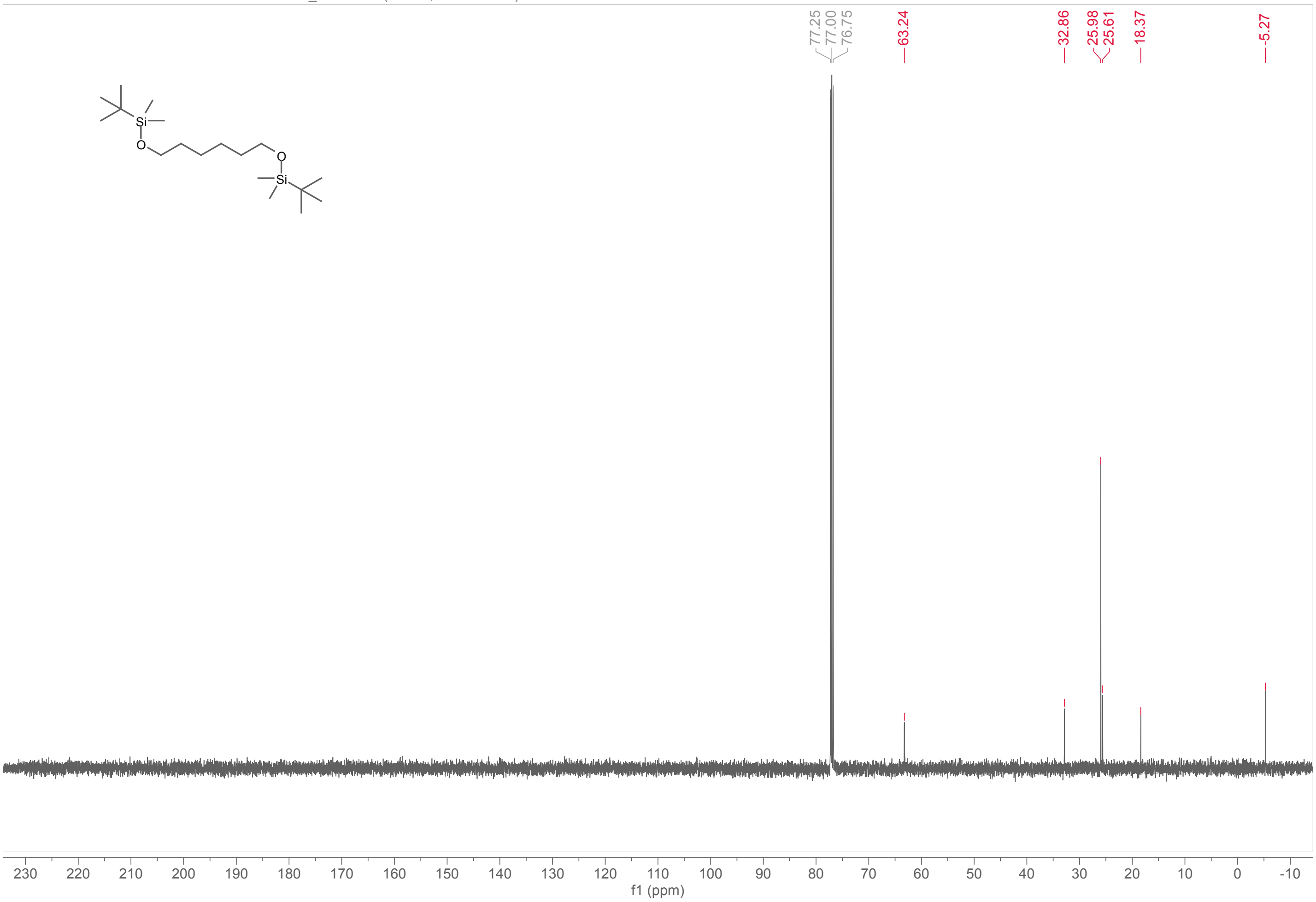

165_1H NMR (CDCl3, 499.94 MHz)

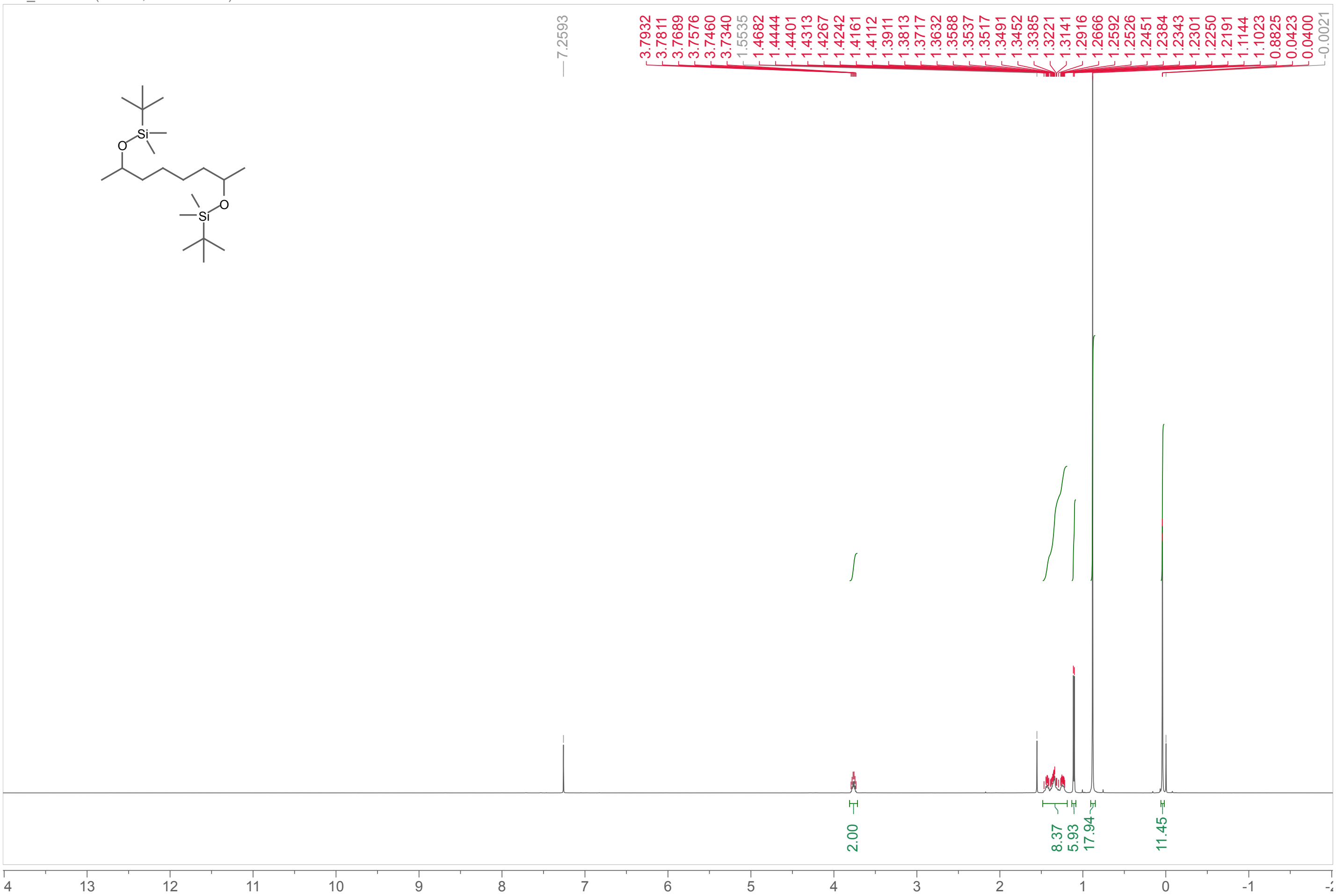


165_13C NMR (CDCl3, 125.72 MHz)

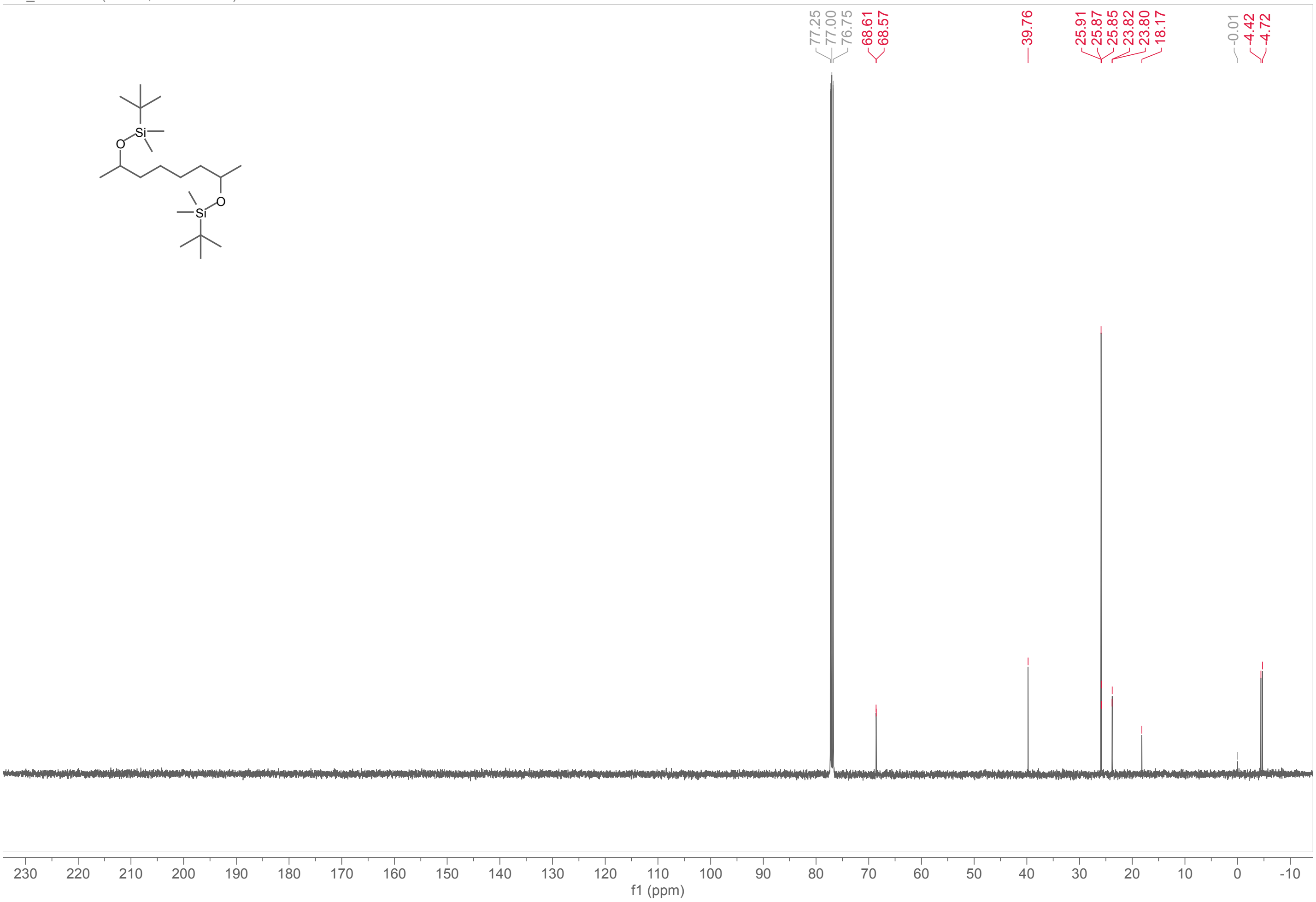

187_1H NMR (CDCl3, 499.94 MHz)

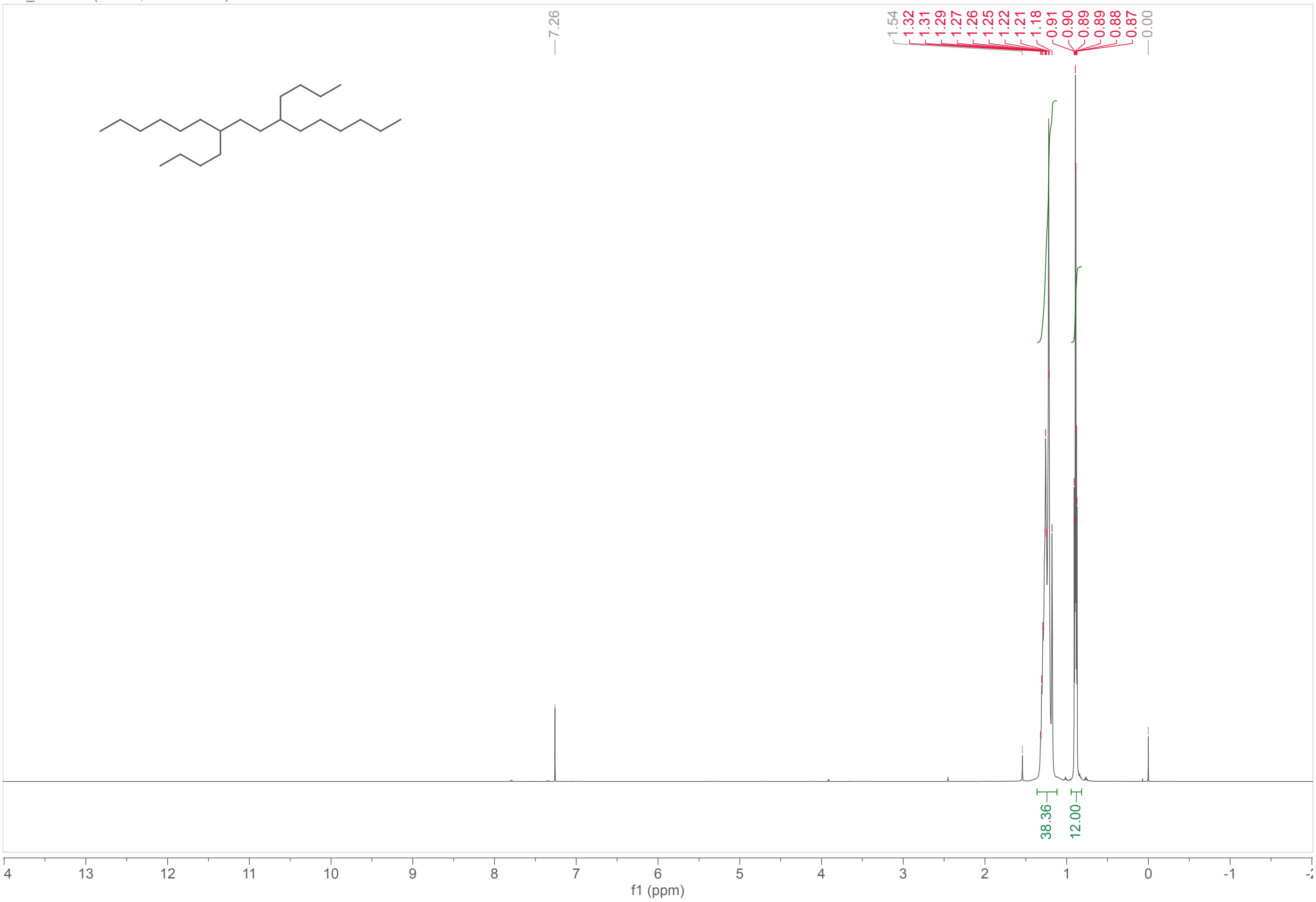


187_13C NMR (CDCl3, 125.72 MHz)

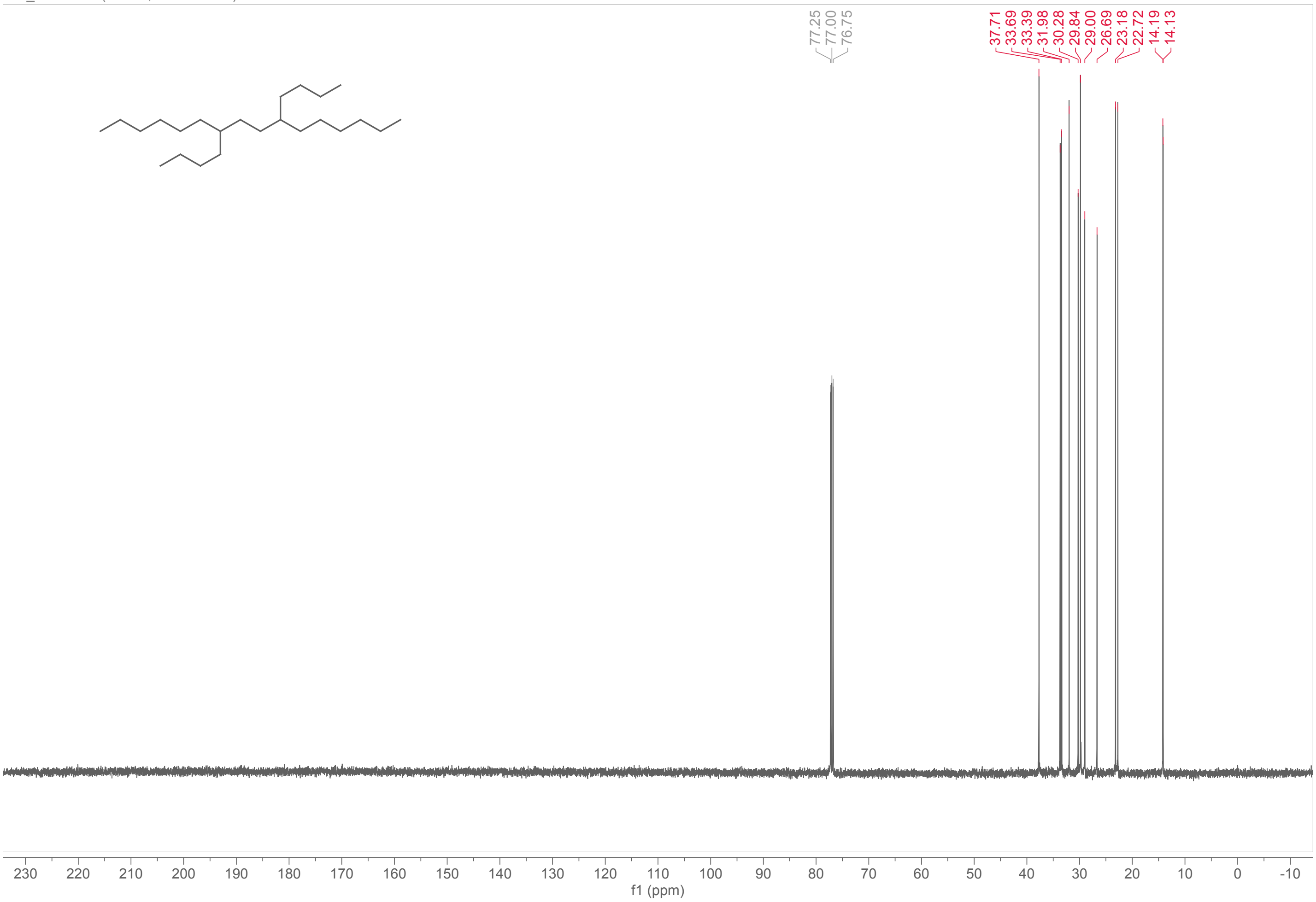

194_1H NMR (CDCl3, 499.94 MHz)

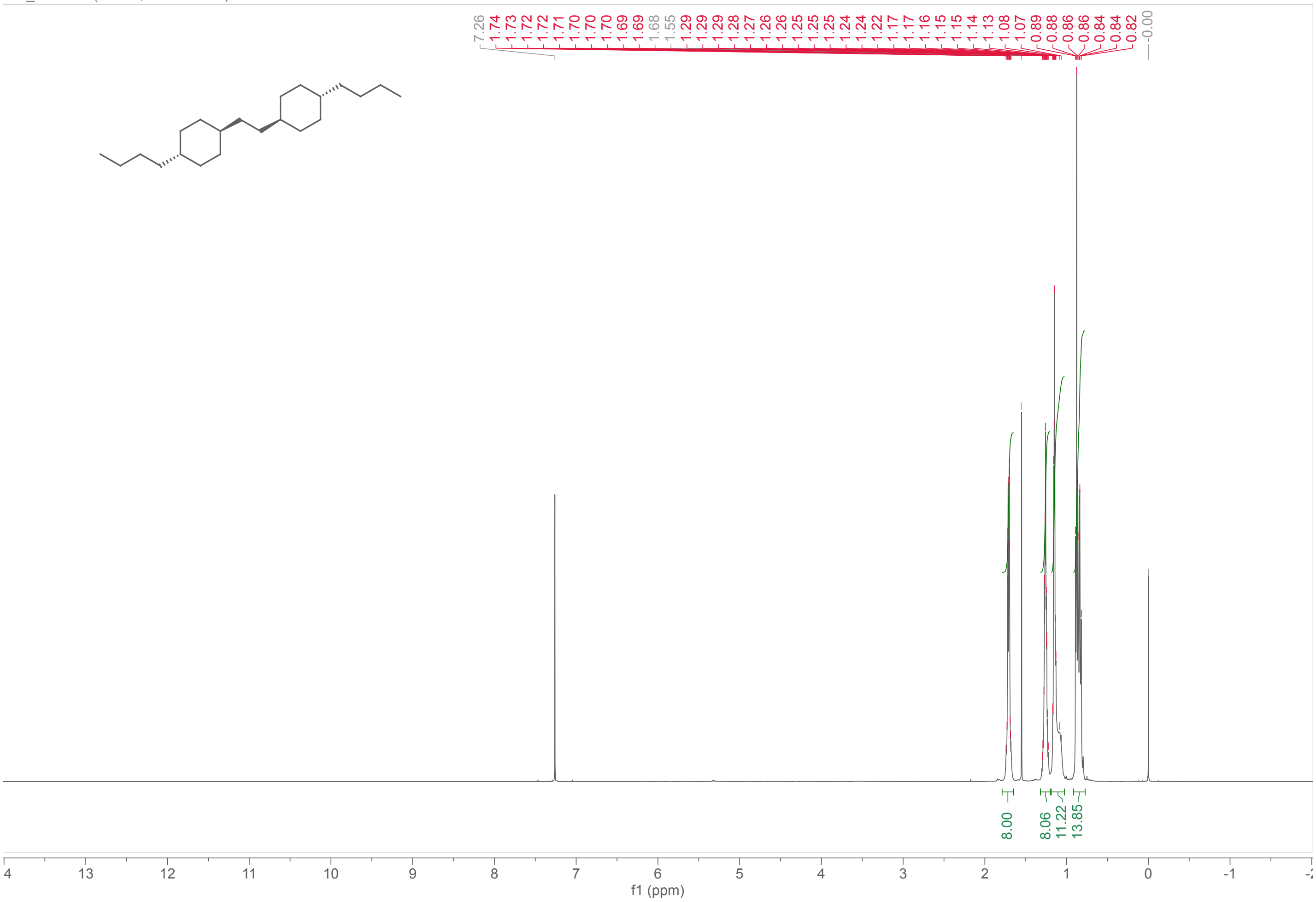


194_13C NMR (CDCl3, 125.72 MHz)

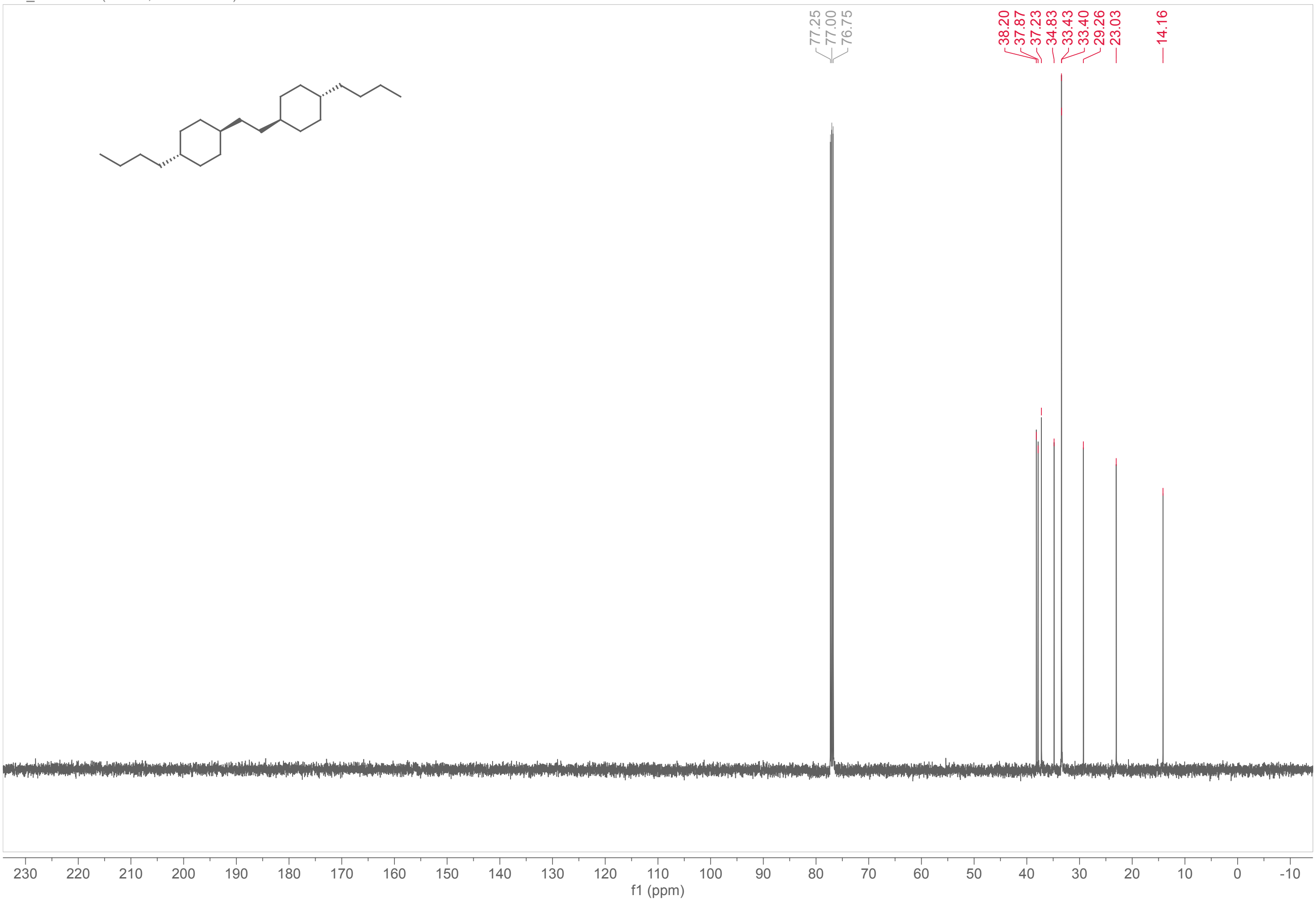

Supplement: Supplementary file 1 [file molecules-24-01458-s001.pdf]
